# Deciphering the Behavioral Response of *Meloidogyne incognita* and *Fusarium oxysporum* Toward Mustard Essential Oil

**DOI:** 10.3389/fpls.2021.714730

**Published:** 2021-08-26

**Authors:** Anirban Dutta, Abhishek Mandal, Aditi Kundu, Monika Malik, Amrendra Chaudhary, Matiyar Rahaman Khan, Veerubommu Shanmugam, Uma Rao, Supradip Saha, Neeraj Patanjali, Rajesh Kumar, Anil Kumar, Sukanta Dash, Pradeep Kumar Singh, Anupama Singh

**Affiliations:** ^1^Division of Agricultural Chemicals, Indian Council of Agricultural Research (ICAR)-Indian Agricultural Research Institute, New Delhi, India; ^2^Division of Nematology, Indian Council of Agricultural Research (ICAR)-Indian Agricultural Research Institute, New Delhi, India; ^3^Division of Plant Pathology, Indian Council of Agricultural Research (ICAR)-Indian Agricultural Research Institute, New Delhi, India; ^4^Division of Design of Experiments, Indian Council of Agricultural Research (ICAR)-Indian Agricultural Statistical Research Institute, New Delhi, India

**Keywords:** essential oil, allyl isothiocyanate, root-knot nematode, molecular docking, GC-MS analysis, nematostasis

## Abstract

Environmental concerns related to synthetic pesticides and the emphasis on the adoption of an integrated pest management concept as a cardinal principle have strengthened the focus of global research and development on botanical pesticides. A scientific understanding of the mode of action of biomolecules over a range of pests is key to the successful development of biopesticides. The present investigation focuses on the *in silico* protein-ligand interactions of allyl isothiocyanate (AITC), a major constituent of black mustard (*Brassica nigra)* essential oil (MEO) against two pests, namely, *Meloidogyne incognita* (Mi) and *Fusarium oxysporum* f. sp. *lycopersici* (Fol), that cause severe yield losses in agricultural crops, especially in vegetables. *The in vitro* bioassay results of MEO against Mi exhibited an exposure time dependent on the lethal concentration causing 50% mortality (LC_50_) values of 47.7, 30.3, and 20.4 μg ml^−1^ at 24, 48, and 72 h of exposure, respectively. The study revealed short-term nematostatic activity at lower concentrations, with nematicidal activity at higher concentrations upon prolonged exposure. Black mustard essential oil displayed excellent *in vitro* Fol mycelial growth inhibition, with an effective concentration to cause 50% inhibition (EC_50_) value of 6.42 μg ml^−1^. In order to decipher the mechanism of action of MEO, its major component, AITC (87.6%), which was identified by gas chromatography–mass spectrometry (GC-MS), was subjected to *in silico* docking and simulation studies against seven and eight putative target proteins of Mi and Fol, respectively. Allyl isothiocyanate exhibited the highest binding affinity with the binding sites of acetyl cholinesterase (AChE), followed by odorant response gene-1 (ODR1) and neuropeptide G-protein coupled receptor (nGPCR) in Mi, suggesting the possible suppression of neurotransmission and chemosensing functions. Among the target proteins of Fol, AITC was the most effective protein in blocking chitin synthase (CS), followed by 2,3-dihydroxy benzoic acid decarboxylase (6m53) and trypsinase (1try), thus inferring these as the principal molecular targets of fungal growth. Taken together, the study establishes the potential of MEO as a novel biopesticide lead, which will be utilized further to manage the Mi–Fol disease complex.

## Introduction

Plant parasitic nematodes and soil-borne pathogens, in particular, pose a serious management challenge for crop growers (Jaiswal et al., 2017). The plant disease complex of wilt fungus (*Fusarium oxysporum*) and root-knot nematode (*Meloidogyne incognita*) severely affects the number of crops (Kassie et al., 2020; Khan and Sharma, 2020). The exact mechanism of infestation of this disease complex is still unknown, though the predisposition phenomenon of susceptibility of *M. incognita*-infested plants to *F. oxysporum* has been largely reported (Back et al., 2002; Khan and Sharma, 2020).

Commonly employed pest management practices in this context involve the usage of pesticides to manage *M. incognita* and *F. oxysporum* individually. New generation molecules such as fluensulfone, fluopyram, fluazaindolizine, and tioxazafen have been reported as effective nematicides (Desaeger and Watson, 2019; Silva et al., 2019; Chen et al., 2020). Furthermore, synthetic fungicides like carbendazim, benomyl, prochloraz, fludioxonil, propiconazole, thiabendazole, bromuconazole, and thiophanate have long been in use for *Fusarium* wilt management (Song et al., 2004; Amini and Sidovich, 2010; Ajilogba and Babalola, 2013). There is, however, a growing concern over synthetics due to their injudicious use, which has led to resistance in pests, toxicity to non-target organisms, residue issues in food and the environment, and other associated health hazards. Globally, many effective synthetic pesticides are facing bans (Wightwick et al., 2010; Chen et al., 2020). Thus, preference is being given to an Integrated Pest Management (IPM) concept that involves using biopesticides as a major component for pest management (Wanjohi et al., 2018).

Several bioactives of botanical origin like neem seed powder (Agbenin et al., 2004; Hadian et al., 2011), dried powders of plant parts (Ghazalbash and Abdollahi, 2013), crude leaf extracts (Sharma and Trivedi, 2002; Haseeb et al., 2005; Khieya et al., 2018; Hajji-Hedfi et al., 2019), and essential oils (Gupta et al., 2011) have been used in an attempt to suppress the nematode-disease complex. In particular, the bio-efficacy of plant-derived volatile aromatic essential oils (EO) against root-knot nematodes and fungal pathogens has been extensively reported (Gupta et al., 2011; Andrés et al., 2012; Park et al., 2017; Sharma et al., 2017; Subedi et al., 2020; Basaid et al., 2021), though the mechanism of their action is still unclear (Rajasekharan et al., 2020).

Essential oils primarily composed of volatile mono- and sesquiterpenoids are distilled from plants. As an exception, the EO derived from mustard seeds (MEO) possesses a distinct chemical composition of non-phenolic, non-terpenoidal volatile phytochemical and allyl isothiocyanate (AITC) (Turgis et al., 2009). Mustard seed essential oil has been reported as a highly effective biofumigant for the management of stored grain pests (Paes et al., 2012). The fumigant action of AITC to control soil-borne diseases in tomato crops has been confirmed by Cao et al. (2006) and Ren et al. (2018). Sporadic reports regarding the potential of AITC to manage plant parasitic nematodes are also available (Zasada and Ferris, 2003; Dhingra et al., 2004). The biofumigation of field soil using mustard plants and seed meal is an indigenous traditional knowledge-based approach for the management of root knot nematodes (Tiyagi et al., 2001). However, neither a ready-to-use MEO-based biopesticide is available nor a quantifiable assessment of its bioefficacy and mode of action as a biocide against either of the pests has ever been attempted. The present study aimed to understand the behavioral responses of *M. incognita* (Mi) and *F. oxysporum* f. sp. *lycopersici* (Fol) toward *in vitro* exposure to MEO and predict mode of action using an *in silico* interaction analysis.

## Materials and Methods

### Chemicals and Reagents

Essential oil from black mustard [*Brassica nigra* (L.) Koch] seeds was purchased from Moksha Lifestyle Products^TM^ (New Delhi, India) and used in the study without further purification. Two surfactants, namely, Triton X-100 and Atlas G5002, were procured from Loba Chemie Pvt. Ltd. (Mumbai, India) and Croda India Company Pvt. Ltd. (Navi Mumbai, India), respectively. Pluronic F-127 powder was purchased from Sigma-Aldrich Chemicals Pvt. Ltd. (Bangalore, India), and potato dextrose agar (PDA) powder, potato dextrose broth (PDB) powder, and resazurin sodium salt were obtained from HiMedia Laboratories Pvt. Ltd. (New Delhi, India). Oil O Red dye was supplied by Alfa Aesar, Thermo Fisher Scientific India Pvt. Ltd. (Mumbai, India). All the solvents used in the study were purchased from Merck Life Science Pvt. Ltd. (New Delhi, India) and were of AR grade.

### Gas Chromatography–Mass Spectrometry Analysis

A gas chromatography (GC) instrument (7890A, Agilent Technologies, Santa Clara, CA, USA) equipped with an HP-5MS column (30 m × 0.25 mm i.d.; film thickness: 0.25 μm), used as the stationary phase, and directly connected to a Triple-Axis HED-EM 5975C mass detector (Agilent Technologies, USA), was used to analyze the MEO. Helium of high purity (99%) was used as a carrier gas with a flow rate of 1 ml min^−1^ and head pressure of 10 psi. A sample volume of 1 μl was injected through an autosampler in a 1:20 split ratio. A run time of total 65.67 min was set for the GC–mass spectrometry (GC-MS) analysis with the oven temperature programmed as follows: initial holding at 40°C for 1 min, increasing at 3°C min^−1^ up to 120°C, holding at 120°C for 2 min, again increasing at 5°C min^−1^ up to 220°C, holding at 220°C for 1 min, and finally increasing at 4°C min^−1^ up to 280°C. Ion source and transfer line temperatures were maintained at 180 and 280°C, respectively. Mass spectra were acquired at full scan mode (50–550 AMU) with electron ionization (EI) at 70 eV. The National Institute of Standards and Technologies (NIST) Mass Spectra Library was used for the identification of compounds by closest matching of mass fragmentation pattern.

### Nematode Bioassay

#### Collection of Nematodes

The culture of Mi was maintained under greenhouse conditions on infected tomato plants (var. Pusa Ruby). During transplantation, the 4-week-old tomato seedlings were inoculated with Mi. After 45 days of transplantation, the egg masses were picked from the galled roots of the infected tomato seedlings and kept for hatching for 5 days at 27 ± 1°C on an assembly comprising a wet soft tissue paper containing aluminum wire gauze and kept on a Petri plate filled with fresh distilled water. Freshly hatched second-stage juveniles (J_2_s) of Mi wriggled through the tissue paper into the clear water in the Petri plate. The J_2_ suspension was collected, and their density was estimated using a stereo-microscope (Leica S8 APO, Leica Microsystems, Wetzlar, Germany).

#### Preparation of MEO Emulsions

Primary stock emulsion (10,000 μg ml^−1^) was prepared in distilled water using Triton X-100 surfactant (4% w/v). Ultrasonication of the stock emulsion was carried out in an ultrasonic bath sonicator (PCI Analytics Pvt. Ltd., Mumbai, India; 250 W; 10–20 min). Test emulsions of 2–200 μg ml^−1^ strength were prepared through a serial dilution technique using the same surfactant solution.

#### Evaluation of Bioactivity Against *M. incognita in vitro*

*In vitro* bioactivity of MEO against Mi was conducted following standard procedure (Kundu et al., 2016). Briefly, aliquots (1 ml) of nematode suspension containing ~120 J_2_s were placed separately in Petri plates (40 mm i.d.). An equal volume (1 ml) of test MEO emulsion of a particular strength (2–200 μg ml^−1^) was dispensed in the individual Petri plate to achieve the 1–100 μg ml^−1^ test concentrations. The Triton X-100 solution (4%, w/v) was used as negative control. The Petri plates were incubated at 27 ± 1°C, and observations were recorded under a stereo-microscope at 2-, 4-, 6-, 24-, 48-, and 72-h intervals. For each treatment and time interval set, six replications were kept. The number of immobilized and freely moving juveniles was recorded at a specified time period (2, 4, 6, 24, 48, and 72 h). A revival test was conducted by transferring the immobilized nematodes with straightened bodies to fresh Petri plates containing 1 ml of distilled water, after 24, 48, and 72 h of treatment exposure. Few drops of 1 M sodium hydroxide (NaOH) solution were then added to the Petri plates containing immobilized juveniles (Chen and Dickson, 2000). The nematodes that instantaneously responded to alkali exposure by changing from straight to curved or hook-shaped posture were considered alive, while the remaining ones that did not respond were considered dead. Corrected mortality (%) of Mi J_2_s was calculated as (Kundu et al., 2016):

$$\begin{array}{l} \text{Corrected mortality }\left(\text{\%}\right)=\left[\left(\text{T}-\text{C}\right)/\left(100-\text{C}\right)\right]\times 100 \end{array}$$

where T = average mortality (%) in treatment and C = average mortality (%) in negative control.

#### Assessment of Infectivity of Immobilized Mi Juveniles

To assess the infectivity potential of nematode juveniles immobilized due to MEO exposure, 4-day-old tomato (var. Pusa Ruby) seedlings were subjected to infection by the treated juveniles in a Pluronic gel medium. The pluronic gel was prepared by dissolving Pluronic F-127 powder (23 g) in distilled water (80 ml) with continuous stirring at 4°C for 24 h (Wang et al., 2009). The solution (15 ml) was poured into Petri plates (60 mm i.d.) containing nine uniformly distributed tomato seedlings. Juveniles exposed to MEO emulsions (5, 10, and 20 μg ml^−1^) for different time periods (1, 6, and 24 h) were taken in Eppendorf tubes (1.5 ml) and centrifuged at 1,685 × g for 5 min. The pellet containing immobilized J_2_s was rinsed twice with distilled water. Twenty-five immobilized J_2_s from each treatment were dispensed separately at the root tip of each of the tomato seedlings kept in the Pluronic gel with the help of a pipette tip. The gel was allowed to set at room temperature, and then, the covered Petri plates were incubated at 27 ± 1°C. The experiment was replicated three times.

After 48 h of inoculation, the seedlings were carefully removed from the gel matrix after liquefying the gel by placing the Petri plates in an ice bath. Roots were bleached with a 4% sodium hypochlorite (NaOCl) solution for 30 s, rinsed five times with distilled water to remove any NaOCl, and finally stained using an acid fuchsin dye (Bybd et al., 1983). The number of nematodes invaded inside the roots was counted under stereo-microscope.

#### Assessment of MEO Permeation Inside Nematode Body

The essential oil from black mustard seeds stock emulsion (100 μg ml^−1^) was prepared in distilled water using Triton X-100 (2%, w/v). During preparation, an oil soluble dye, namely, Oil O Red, was mixed with the emulsion to prepare a 50 μg ml^−1^ concentration, followed by ultrasonication at a bath sonicator (PCI Analytics Pvt. Ltd., Mumbai, India) for 20 min. The stock emulsion was diluted with the surfactant solution to obtain a test emulsion comprising 10 μg ml^−1^ of MEO and 5 μg ml^−1^ of Oil O Red. A control emulsion containing only 5 μg ml^−1^ of Oil O Red was also prepared in a similar way. Both the test and control emulsions (2 ml) were placed separately in Petri plates (40 mm i.d.), and a 1 μl suspension of Mi J_2_s was dispensed in each Petri plate. After incubating the Petri plates at 27 ± 1°C for 6 h, nematodes (immobilized and alive) from both emulsions were picked up on glass slides, heat-killed, and washed with distilled water. The stained nematodes were then observed using epifluorescence on a Zeiss Axiophot Photomicroscope (Zeiss International, Oberkochen, Germany). The fluorescence intensity of the captured images was quantified using the NIH software ImageJ (version 1.48). The corrected total cell fluorescence (CTCF) was calculated using the following formula (Park et al., 2020):

$$\begin{array}{l} \text{CTCF}=\text{Integrated Density}\\ -(\text{Area of selected cell}\times \text{Mean fluorescence of}\\ \text{ background readings}) \end{array}$$

### Antifungal Bioassay

#### Fungal Culture

A highly virulent Fol strain, TOFU-IHBT, available at the laboratory collections of the Division of Plant Pathology, ICAR-Indian Agricultural Research Institute, New Delhi, was used as the test pathogenic fungus (Sidharthan et al., 2018). The fungal culture was activated by sub-culturing on a PDA medium and incubation at 27 ± 1°C.

#### Poisoned Food Assay

The antifungal activity of MEO was tested against the pathogenic fungus by a poisoned food technique as described earlier with slight modifications (Dutta et al., 2020). Briefly, PDA powder (39 g) was suspended in 1 L of distilled water and autoclaved at 120°C for 30 min. A stock emulsion of MEO (10,000 μg ml^−1^) was prepared in distilled water using Atlas G5002 surfactant (2%, w/v). The stock emulsion was aseptically mixed with a measured volume of PDA slurry to obtain the desired test concentrations (1–1,000 μg ml^−1^). The medium was poured into sterile Petri plates (45 mm i.d.) and allowed to solidify. Mycelial discs (10-mm diameter) were punched from a 7-day-old culture of TOFU-IHBT and placed at the center of each Petri plate. Plates with only the PDA medium (without MEO) were also inoculated with the pathogen and kept as a negative control. Nativo 75 WG (25% trifloxystrobin + 50% tebuconazole; Bayer Crop Science Ltd., Mumbai, India) was used as positive control. Three replicates were maintained for each treatment, and the Petri plates were incubated at 27 ± 1°C. Mycelial growths were measured diametrically when the growth in the negative control plates reached full. Mycelial growth inhibition was calculated using the following formula (Kundu et al., 2013):

$$\begin{array}{l} \text{Inhibition of growth }\left(\text{\%}\right)=\left[\left(\text{C}-\text{T}\right)/\text{C}\right]\times 100 \end{array}$$

where C is the average of three replicates of mycelial growth (diameter in mm) in the negative control, and T is the average of three replicates of mycelial growth (diameter in mm) of the treated plates.

#### Resazurin Microtiter-Plate Assay

The minimum inhibitory concentration (MIC) of MEO was determined using a modified resazurin microtiter-plate assay method (Sarker et al., 2007). Briefly, 80 μl of PDB media and 10 μl of a resazurin indicator solution (0.7%) were taken in each well of a sterile 96-well plate. The MEO stock emulsion was poured into the plates by a 2-fold serial dilution technique in descending order in such a way that each well-received 50 μl of the test emulsion. Finally, 10 μl of fungal spore suspension was added to each well to achieve a concentration of ~1 × 10^5^ cfu ml^−1^ and a total volume of 150 μl. A set of controls was also maintained, viz., C_1_: sterile control without addition of test emulsion and fungal suspension, complemented with 60 μl of PDB media instead; C_2_: negative control without the addition of the test emulsion, complemented with 50 μl of PDB media instead; C_3_: a negative control with a surfactant solution (Atlas G5002, 2% w/v) instead of the test emulsion; and C_4_: positive control with the addition of Nativo suspension instead of the test emulsion. The plates were prepared in triplicates and incubated at 27 ± 1°C for 48 h. A visual assessment of the color changes from purple to pink (or colorless) was recorded as positive. The lowest concentration at which color change occurred was noted as the MIC. Thus, the MIC value was interpreted as the lowest concentration of the test emulsion that did not show any fungal growth.

### *In silico* Molecular Modeling

Allyl isothiocyanate, the major volatile constituent of MEO, was subjected to *in silico* molecular docking for seven putative target protein receptors of Mi to understand the mechanism of its nematocidal action. Similarly, an *in silico* ligand–protein interaction analysis of the oil was studied with eight putative target protein receptors of Fol.

#### Selection of Proteins and Protein Preparation

Seven target protein receptors of Mi (Supplementary Table 1) reported by our group in a similar molecular docking study on essential oils (Kundu et al., 2021), namely, cytochrome-c oxidase subunit 1, acetyl cholinesterase (AChE), heat shock protein 90 (Hsp90), odorant response gene-1 (ODR1), odorant response gene-3 (ODR3), neuropeptide G-protein coupled receptor (nGPCR), and CLAVATA3/ESR (CLE)-related protein, have also been employed in this study.

Similarly, eight proteins of Fol, namely, trypsinase (1try), endoglucanase (4ovw), cutinase (5ajh), xylanase (5jrm), Avr2 effector protein (5od4), feruloyl esterase (6fat), 2,3-dihydroxy benzoic acid decarboxylase (6m53), and chitin synthase (CS), were selected. Trypsinase is suspected to be part of the invasion strategy of some pathogenic fungi; 4ovw breaks down the glucan present in the plant cell wall; 5ajh hydrolyzes cutin; 5jrm breaks down the xylan backbone, thus playing a crucial role in xylan depolymerization; 5od4 employs effector protein Avr2 to manipulate the host plant; 6fat cleaves ester crosslinks between hydroxycinnamic acids and arabinoxylans; 6m53 catalyzes the non-oxidative decarboxylation of 2,3-dihydroxybenzoic acid to catechol; and fungal CSs are integral membrane-bound proteins that participate in the biosynthesis of the cell wall and are important for hyphal growth and differentiation.

Secondary structures of the selected Mi protein sequences were homology modeled based on the protein sequences downloaded from the National Center for Biotechnology Information (NCBI) GenBank and Universal Protein Resource (UniProt) databases and aided by the existing homologous templates from the Research Collaboratory for Structural Bioinformatics Protein Data Bank (RSCB PDB). In the case of Fol, the structures of all the target protein molecules except CS were downloaded from the RCSB PDB and conserved during the process. The CS protein sequences were taken from the UniProt database. The Basic Local Alignment Search Tool (BLAST) servers (http://blast.ncbi.nlm.nih.gov) were used to search and annotate the molecular and biological functions of the query sequence. The NCBI BLAST was used with the PDB database to identify the templates for modeling the secondary structures of the query sequences. The Modeler program (version 9.24) was used for the homology modeling of the three-dimensional structures of all the target proteins.

#### Ligand and Receptor Preparation

Ligand (AITC) molecular structure was downloaded as an .sdf file from the PubChem database (pubchem.ncbi.nlm.nih.gov), minimized in the ChemDraw Ultra 11.0 program using an MM2 force field, and customized using the Dock prep tool of AutoDock Vina. Hydrogen molecules were added and the Dunbrack rotamer library was used to replace the incomplete side chains (Dunbrack, 2006). Charges were computed using ANTECHAMBER with AMBER ff14SB charges allotted to standard residues and Gasteiger charges to other residue types. The same tools were used to prepare the receptor molecules. The quality of the homology-modeled proteins was accessed using ProSA-Web for the statistical *Z*-score (Supplementary Figure 1A), while the Ramachandran Plot for determining the residual outliers was accessed using the PROCHECK program (Supplementary Figure 1B). The quality of the modeled proteins was found to be thermodynamically feasible in nature with the desired substructural stability.

#### Molecular Docking and Simulation

The protein structures were converted to the .pdbqt format through the AutodockTools 1.5.7rc1 software (Morris et al., 2009). Using a 30 Å × 30 Å × 30 Å (x, y, z) grid box, which was continually moved 10 Å in the x, y, and z directions to cover the three-dimensional structures of the enzymes, the target proteins were docked to the major constituent of MEO using the AutoDock Vina (version 1.1.2) software (Trott and Olson, 2010). Except for the exhaustiveness parameter that was set to 32, all parameters were used with the default values.

Based on docking score, binding affinity, and interacting residues, the most favored docking conformations between the target proteins and the ligand were ranked. The Discovery Studio Visualizer (version 4.1) was used for the two-dimensional representation of the interactions.

### Statistical Analysis

Data were analyzed using the open-source statistical program JASP (Version 0.14.1). The significance of the differences between variables was tested using a one-way ANOVA. The means were compared using Duncan's multiple range test. Statistical significance was determined at *p* < 0.05. Percent mortality and percent inhibition data were subjected to a probit analysis using the Polo Plus software to determine lethal (LC_50_ and LC_90_, expressed in μg ml^−1^) and effective concentrations (EC_50_ and EC_90_, expressed in μg ml^−1^).

## Results

### Chemical Composition of MEO

**Figure 1:**
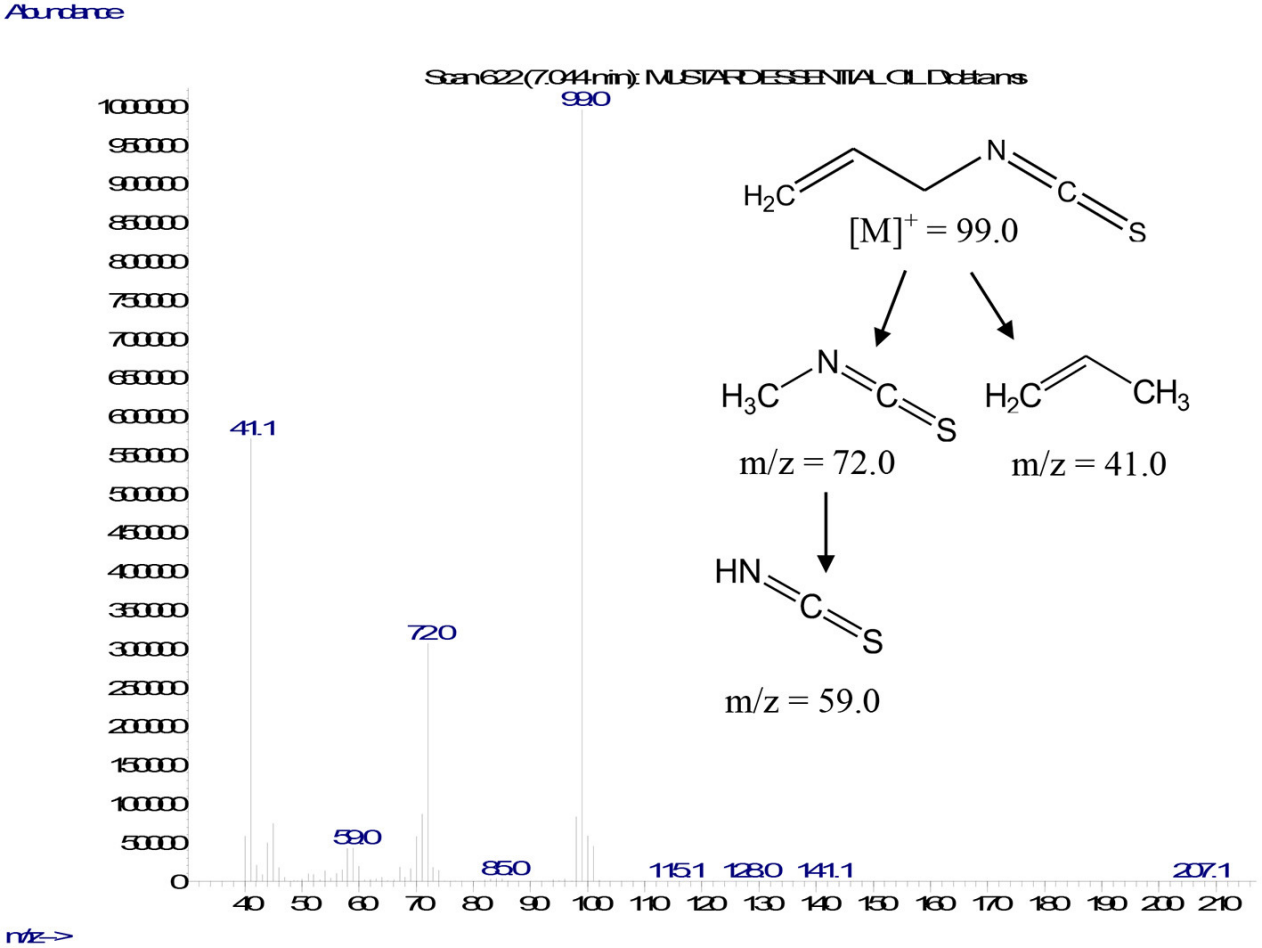
Mass fragmentation pattern of AITC.

A gas chromatography–mass spectrometry analysis of MEO revealed the sole presence of AITC, which was confirmed by its retention indices and mass fragmentation pattern, contributing 87.6 ± 1.9% relative content (mean value of three replicates) of MEO. The mass fragmentation pattern of AITC showed a characteristic molecular ion peak at m/z 99 along with daughter fragment ion peaks at m/z 72, 59, and 41, which appeared due to either the sequential loss of ethene and propene or thiocyanate moieties, respectively (Figure 1).

### Behavioral Response of Nematodes

#### *In vitro* Bioactivity

**Figure 2:**
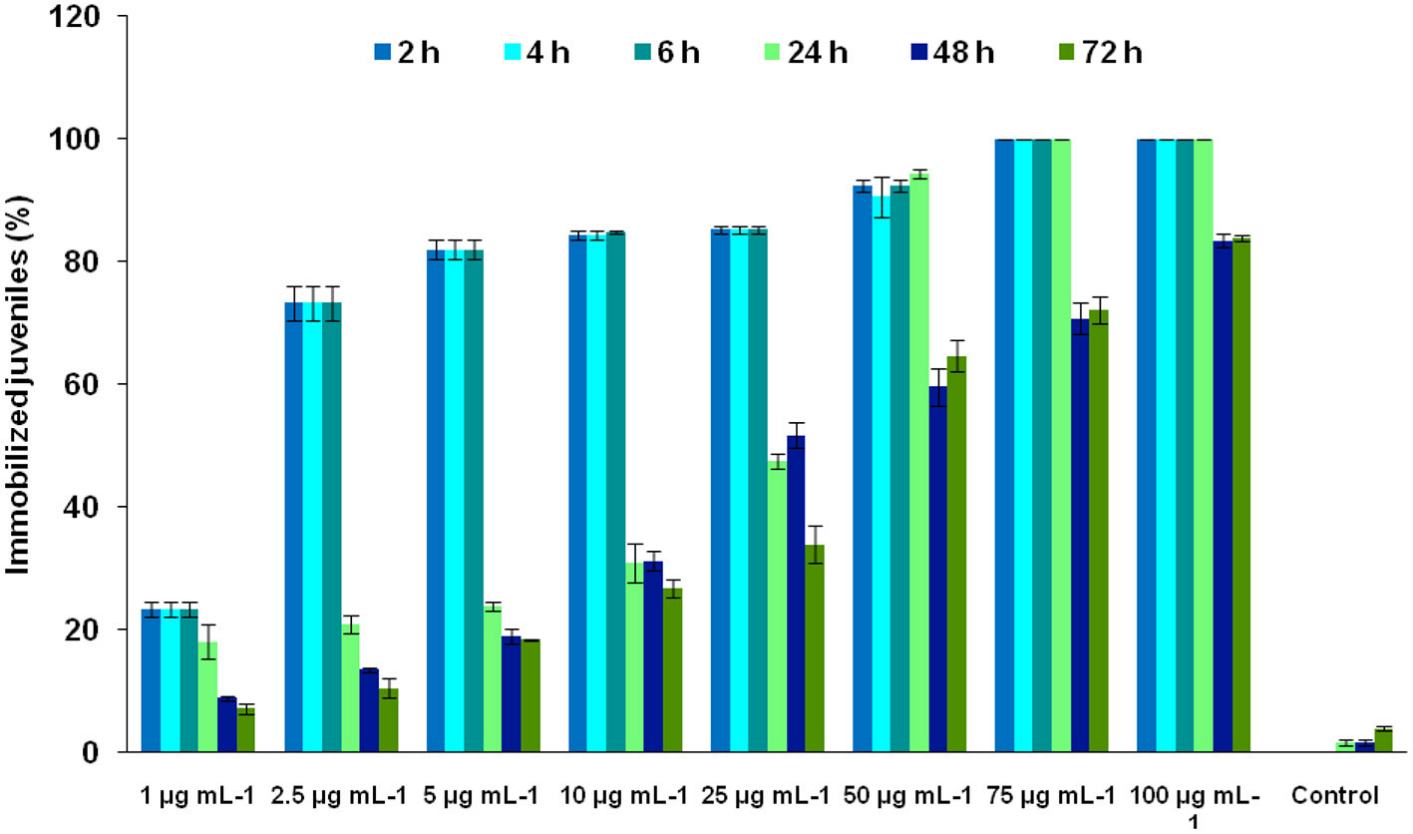
Effect of the concentration of MEO and exposure time on the immobilization of *M. incognita* J_2_s.

The bioassay of MEO against J_2_s of Mi led to novel observations. At lower concentrations, MEO showed nematostatic action. The juveniles became immobilized within 2 h of exposure. An interesting two-way trend was observed. With an increase in MEO concentration (1–100 μg ml^−1^), the nematostatic effect increased, while prolonged incubation at a particular concentration of MEO led to the revival of immobilized juveniles, irrespective of test concentration. As depicted in Figure 2, exposure to 1 μg ml^−1^ concentration for 2 h immobilizes 23.41 ± 1.29% J_2_s, which also increases to 73.25 ± 2.74% at 2.5 μg ml^−1^ concentration. With a further increase in concentration of MEO, the number of immobilized juveniles increased; at 75 μg ml^−1^ concentration and above, all the juveniles were immobilized. The response trend did not change much up to 6 h of exposure at any of the test concentrations. However, after 24 h, the number of immobilized juveniles started decreasing. After 24, 48, and 72 h of exposure at 1 μg ml^−1^ concentration, 18.11 ± 2.84, 8.9 ±.48, and 7.23 ±.93% of the incubated nematodes remained immobilized, respectively. A similar trend was observed for other concentrations up to 25 μg ml^−1^. Though the immobilization effect was prolonged for up to 24 h for concentrations beyond 50 μg ml^−1^, as the percent of immobilized juveniles remained almost same, the revival took place at 48 h. At 75 and 100 μg ml^−1^ concentrations, 100% immobilization occurred up to 24 h, with the values reduced to 70.77 ± 2.65% and 83.45 ± 1.11%, respectively, after 48 h of exposure.

**Figure 3:**
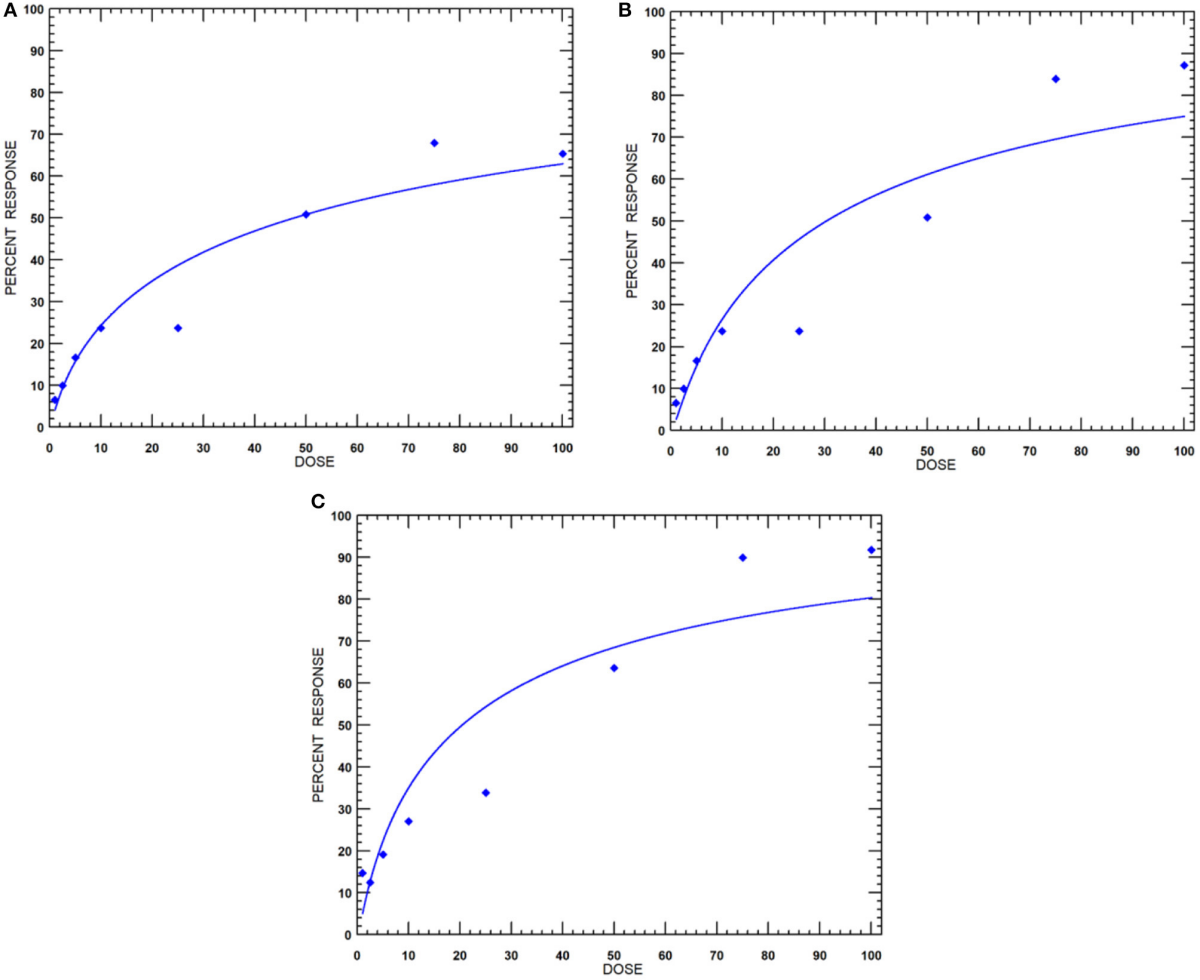
Dose-response curves of MEO against *M. incognita* at incubation periods of **(A)** 24, **(B)** 48, and **(C)** 72 h.

The nematicidal effect of MEO was observed only after 24 h of exposure. The revival test with 1 M of NaOH was used to ascertain the dead nematodes. The relative mortality with an increase in concentration of MEO at 24, 48, and 72 h of exposure is depicted in Figure 3. The lethal concentration causing 50% mortality (LC_50_) values at 24, 48, and 72 h were found to be 47.7, 30.3, and 20.4 μg ml^−1^, respectively (Table 1).

**Table 1:** Probit analysis of the mortality data of *Meloidogyne incognita* to mustard essential oil (MEO) at different time intervals.

**Time (h)**	**LC_**50**_ (μg mL^**−1**^)**	**95 % confidence limit (μg mL** ^**−1**^ **)**		**LC_**90**_ (μg mL^**−1**^)**	**95 % confidence limit (μg mL** ^**−1**^ **)**		**Slope ± SE**	**Intercept ± SE**	**(χ^**2**^)**
		**Lower**	**Upper**		**Lower**	**Upper**			
24	47.7	30.1	91.7	853.2	320.2	5169.0	1.02 ± 0.07	−1.72 ± 0.11	17.24
48	30.3	16.0	74.0	294.5	106.3	3731.0	1.30 ± 0.08	−1.92 ± 0.12	54.55
72	20.4	11.4	40.6	222.6	75.8	4048.2	1.23 ± 0.08	−1.62 ± 0.10	67.16

#### Infectivity of Immobilized Nematodes

**Figure 4:**
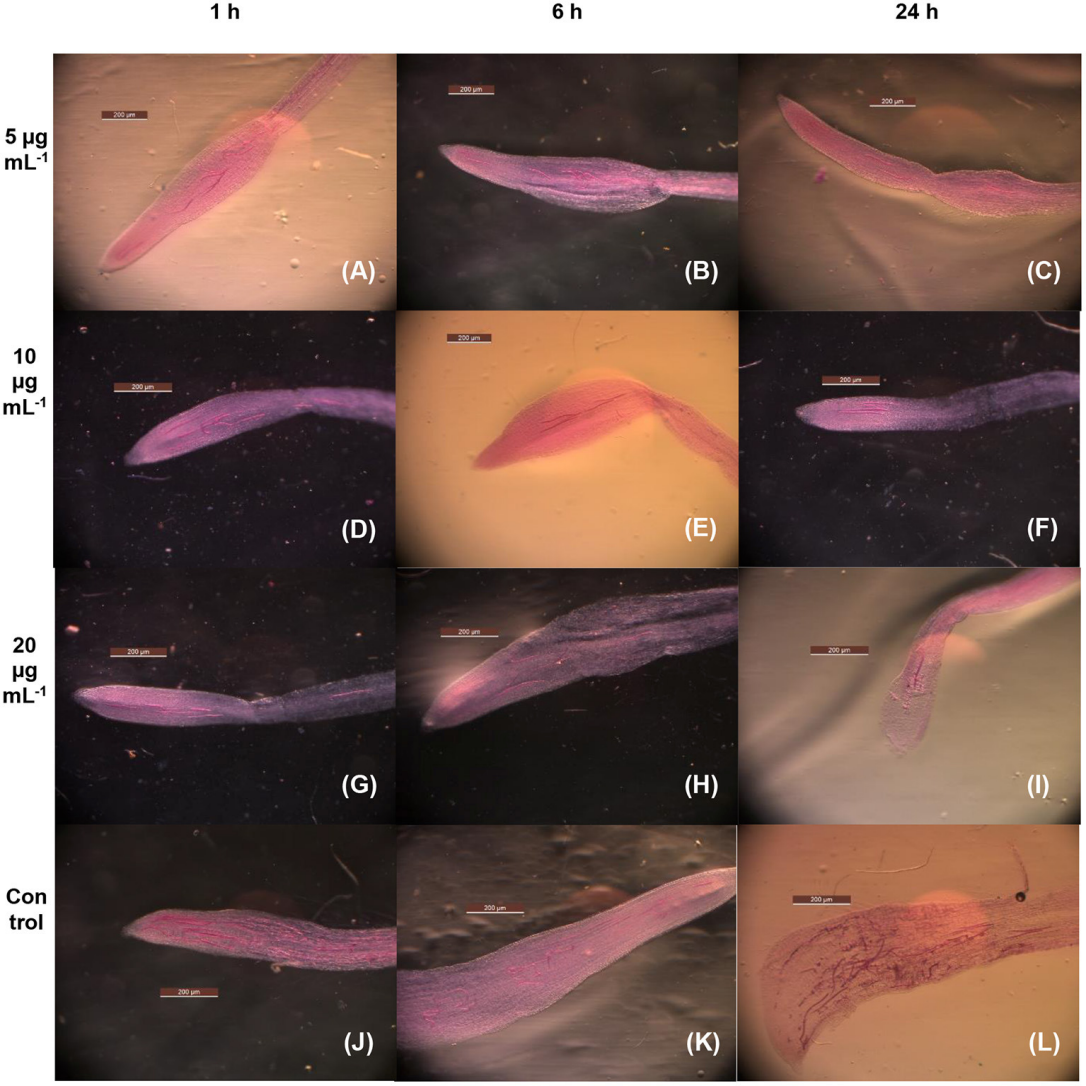
Microscopic images of tomato roots for the infectivity of MEO-treated *M. incognita* exposed at 5 μg mL^−1^ for 1 h **(A)**, 5 μg mL^−1^ for 6 h **(B)**, 5 μg mL^−1^ for 24 h **(C)**, 10 μg mL^−1^ for 1 h **(D)**, 10 μg mL^−1^ for 6 h **(E)**, 10 μg mL^−1^ for 24 h **(F)**, 20 μg mL^−1^ for 1 h **(G)**, 20 μg mL^−1^ for 6 h **(H)**, 20 μg mL^−1^ for 24 h **(I)**, control for 1 h **(J)**, 6 h **(K)**, and 24 h **(L)**.

Table 2 depicts the percent immobilization of juveniles exposed to the MEO test concentrations of 5, 10, and 20 μg ml^−1^ for 1-, 6-, and 24-h periods. Compared with untreated control (55%), the 43.6, 33.8, and 20.2 % J_2_s out of the total population of those exposed to 5, 10, and 20 μg ml^−1^ MEO, respectively, for 1 h could infect tomato roots. The longer period of exposure to MEO at a particular concentration led to a decrease in the infectivity potential of the treated juveniles, as is made evident by the data presented in Table 2. The concentration and infectivity potential of MEO in juveniles clearly exhibited an inverse relationship. Compared to the 71% in untreated control, merely 11.3% of the juveniles exposed to MEO (20 μg ml^−1^) were able to invade the roots. The photomicrographs in Figure 4 confirmed the observed time and dose-dependent response of the nematode. The hitherto unreported observations suggested that, although the MEO-immobilized nematodes managed to survive, their root infectivity potential was severely affected.

**Table 2:** Infectivity of *M*. incognita second stage juveniles (J_2_s) exposed to different concentrations of MEO for different time intervals.

**Concentration of MEO (μg mL^**−1**^)**	**% immobilized J**_**2**_ **s**			**% revived J**_**2**_ **s infecting roots**		
	**1 h**	**6 h**	**24 h**	**1 h**	**6 h**	**24 h**
5	81.99 ± 1.64^b^	81.99 ± 1.64^b^	23.98 ± 0.74^d^	43.56 ± 18.04^ab^	32.22 ± 16.72^b^	24.22 ± 14.22^b^
10	84.39 ± 0.71^b^	84.81 ± 0.13^bc^	31.00 ± 3.20^c^	33.78 ± 15.60^bc^	23.11 ± 9.36^bc^	16.44 ± 9.49^b^
20	85.43 ± 0.79^b^	86.77 ± 0.11^c^	40.74 ± 3.38^b^	20.22 ± 12.68^c^	13.56 ± 10.37^cd^	11.33 ± 9.43^b^
Control^*^	0.13 ± 0.25^a^	0.42 ± 0.48^a^	0.42 ± 0.48^a^	55.00 ± 20.33^a^	67.33 ± 19.06^a^	71.00 ± 16.98^a^

*Tukey's HSD test was performed to check the significant differences among the treatment means within a column. Different alphabets within a column suggest significant differences at 95% confidence limit*.

* *In case of control, nematodes that were alive were transferred to a Pluronic gel plate for an infectivity study*.

#### Permeation of MEO in Nematode Body

**Figure 5:**
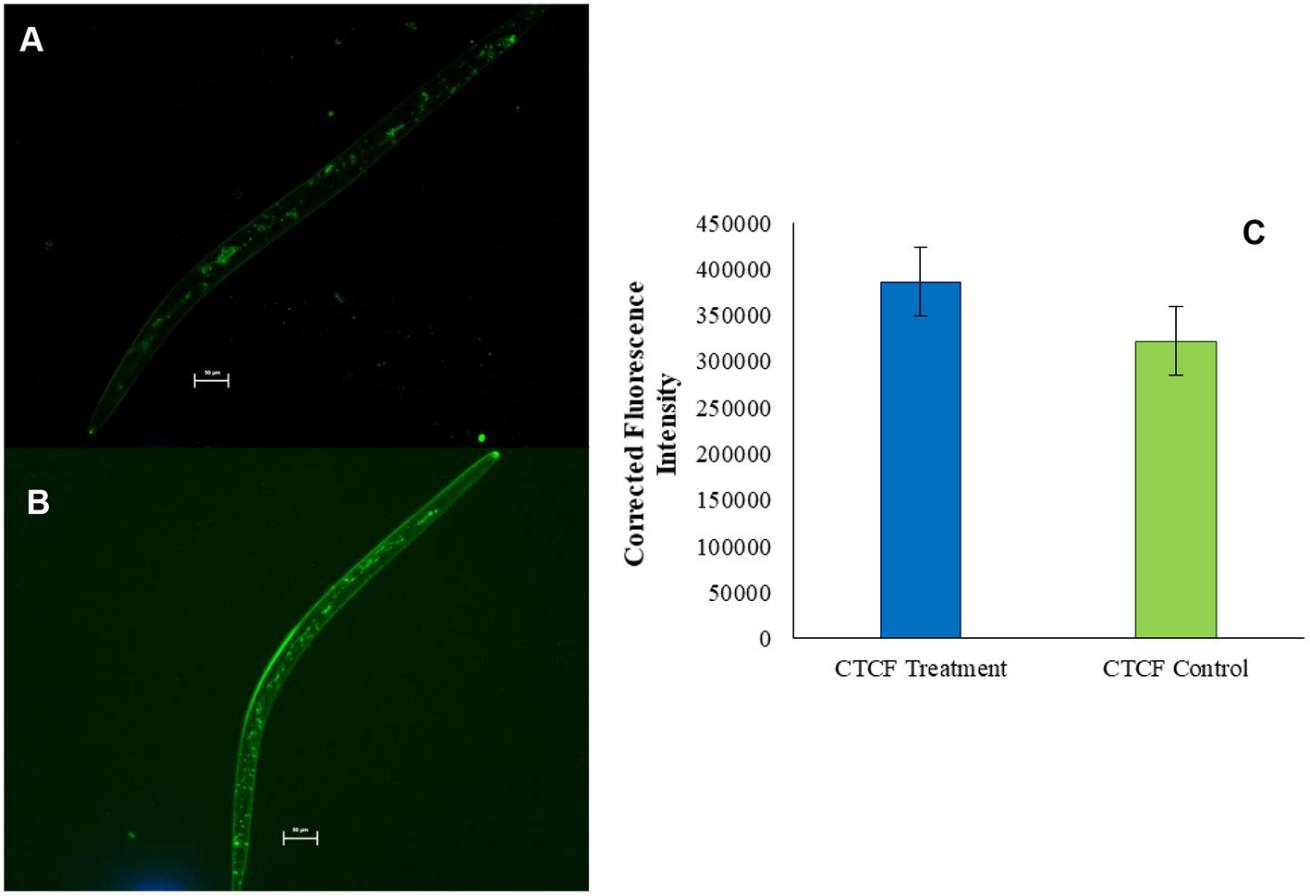
Fluorescence microscopic images of *M. incognita* stained with Oil Red O **(A)** and Oil Red O mixed in MEO **(B)**; comparison of the fluorescence intensities of the two samples **(C)**.

Oil O Red, being an oil soluble dye, stained the lipid reserve of nematode body. Second-stage juveniles exposed to the control emulsion containing dye only (5 μg ml^−1^) were found to be alive. Fluorescence microscopy suggested that the dye was able to penetrate the nematode body and stain the natural fat reserve of organism (Figure 5A). However, when Mi juveniles were exposed to the test emulsion containing both MEO (10 μg ml^−1^) and dye (5 μg ml^−1^), almost 100% immobilization coupled with significant enhancement of the intensity of Mi fluorescence was observed within 6 h (Figure 5B). The CTCF was significantly higher in MEO emulsion-treated nematodes as compared to control (Figure 5C).

### Antifungal Action

**Figure 6:**
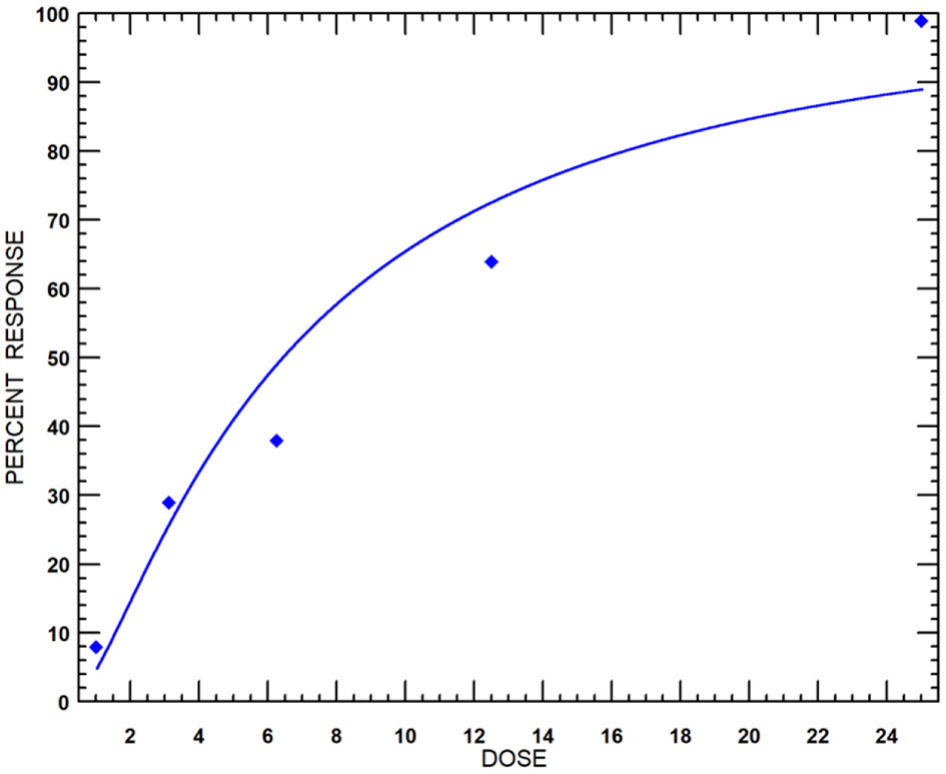
Dose-response curves of MEO against *F. oxysporum* f. sp. *lycopersici* isolate TOFU-IHBT.

Black mustard essential oil exhibited 100% mycelial growth inhibition up to 25 μg ml^−1^ concentration. The effect, however, was relatively less at lower concentrations (Supplementary Figure 2). The probit analysis and the dose–response curve (Figure 6) revealed the effective concentration to cause 50% inhibition (EC_50_) value of MEO to be 6.42 μg ml^−1^. This finding bears significance in the context of the 42.5% mycelial growth inhibition exhibited by Nativo (positive control; trifloxystrobin + tebuconazole) at 50 μg ml^−1^ concentration.

**Figure 7:**
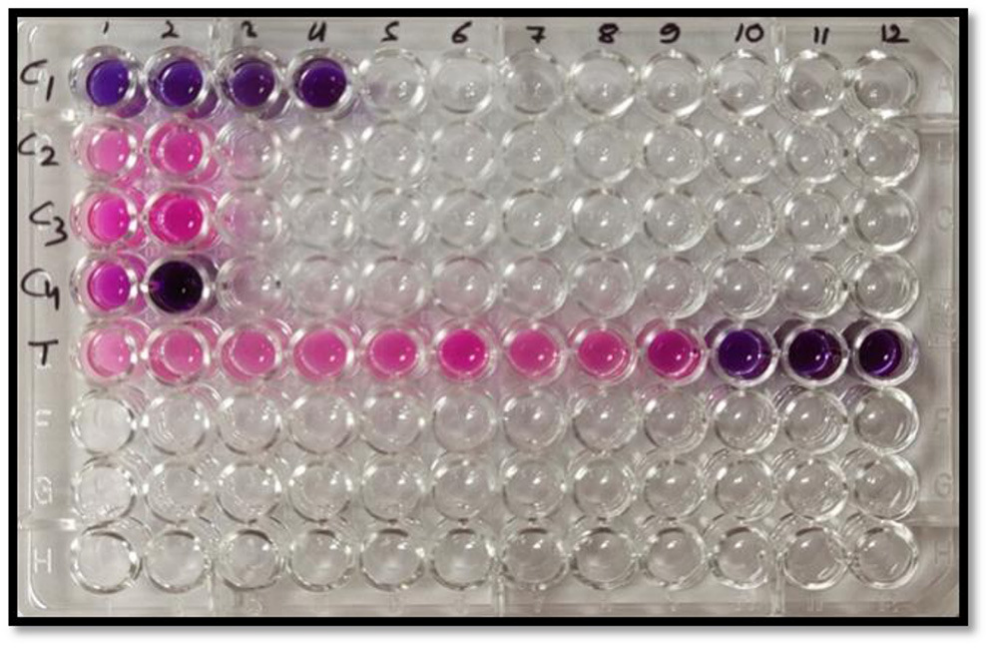
Representative plate of the resazurin assay (pink color indicates growth and blue color indicates no growth); C_1_: sterile control (broth + indicator); C_2_: negative control (broth + indicator + fungus); C_3_: negative control (broth + indicator + fungus + surfactant solution); C_4_: positive control (broth + indicator + fungus + Nativo in two concentrations); T (broth + indicator + fungus + test emulsion in serial dilution with increasing concentration in wells 1–12).

The resazurin microtiter-plate assay was conducted to further confirm these observations and determine the MIC at which no fungal cell remained viable. This simple, rapid, robust, yet sensitive method is very effective to give a response to the presence of any viable cell by changing the blue color of the oxidation-reduction indicator resazurin to pink due to the action of oxidoreductase enzymes present in the viable cells. Figure 7 depicts complete change of color from blue to pink in negative controls, C_2_ (broth + indicator + fungus) and C_3_ (broth + indicator + fungus + surfactant solution), unlike sterile control (C_1_), which did not show any change in color. In MEO treatments, MIC, the minimum concentration at which color change occurred, was 256 μg ml^−1^ corresponding to the treatment T9. In positive control (C4) comprising Nativo, the color change was observed at 1,024 μg ml^−1^.

### Molecular Docking of AITC Against Mi and Fol Target Proteins

Seven target receptor proteins of Mi were screened against AITC, the identified major component of MEO. The docking scores of the screened targets presented in Table 3 range between −14.32 and −21.42. Accordingly, the target proteins could be arranged as AChE > nGPCR > ODR1 > Hsp90 > CLAVATA3/ESR (CLE)-related protein > ODR3 > Cytochrome-c oxidase subunit 1, in descending order of the computed docking score. The three representative receptor targets, i.e., AChE, nGPCR, and ODR1, were thus chosen for further refined molecular docking studies with AITC.

**Table 3:** *In silico* docking results of allyl isothiocyanate (AITC) against *M. incognita* target proteins.

**Target protein**	**Dock score**	**Binding energy (kcal mol^**−1**^)**
Cytochrome c oxidase subunit 1	−14.32	-
AChE	−21.42	−10.5258
Hsp90	−15.63	-
ODR1	−15.77	−8.9653
ODR3	−14.55	-
Neuropeptide GPCR	−19.01	−6.5287
CLAVATA3/ESR (CLE)-related protein	−15.07	-

**Figure 8:**
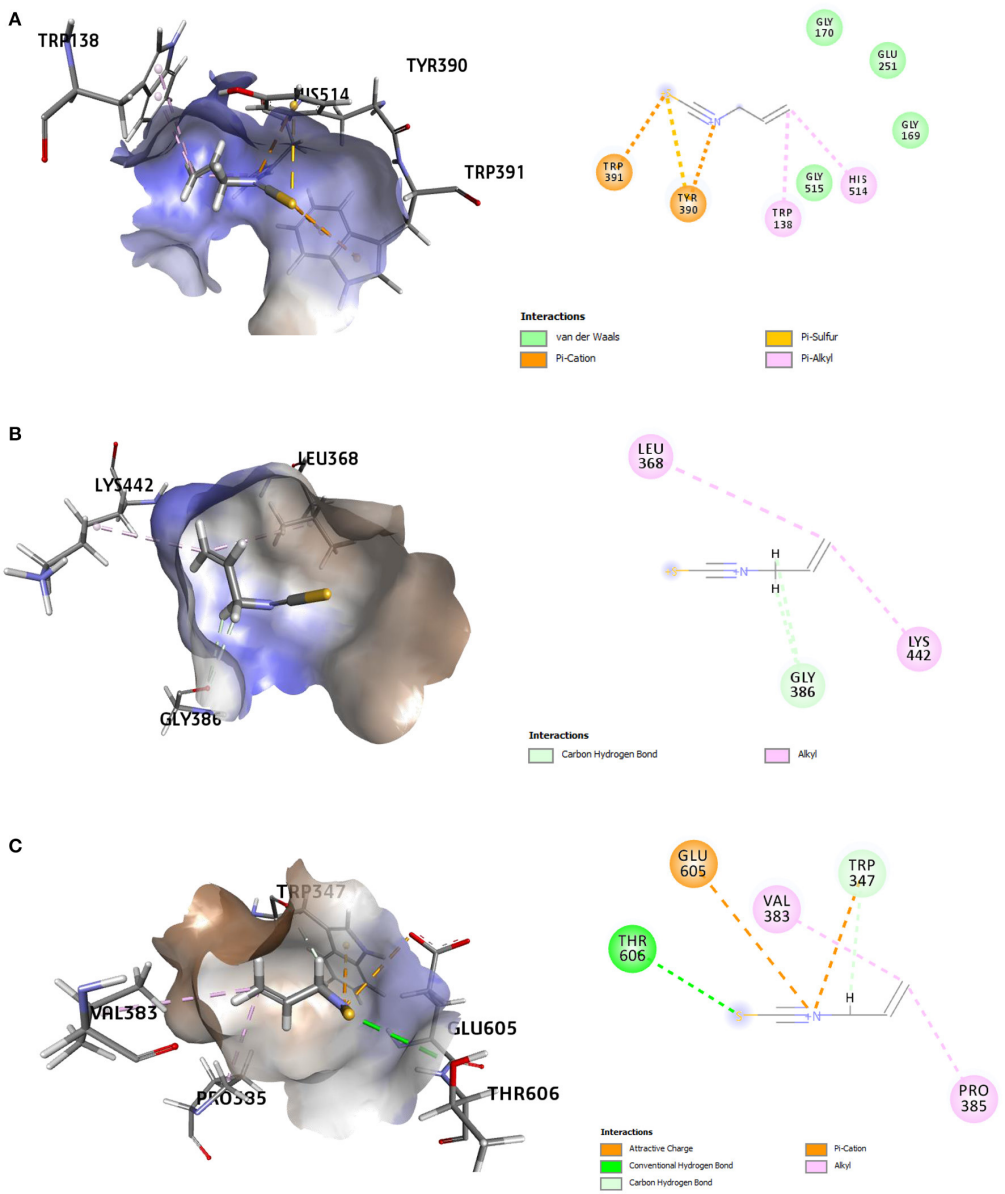
3D and 2D representations of the AITC-bound target sites of **(A)** acetyl cholinesterase (AChE), **(B)** neuropeptide (G-protein coupled receptor) GPCR, and **(C)** odorant response gene-1 (ODR1).

The refined docked complexes of the three targets and AITC were studied, and their binding affinities were computed (Table 3). The most favorable docking and highest binding affinity value observed in the case of the AITC-AChE complex may be attributed to the electrostatic bonding (π-cation category) between the partial positive charge on the nitrogen of –N–C=S moiety of AITC and the π-orbital electrons of the aromatic ring of TYR390 residue (Figure 8A). Moreover, three non-classical type interactions, viz., π-cation type of electrostatic interaction, π-donor hydrogen bonding, and the π-sulfur bond between sulfur (partial negative charge) atom of AITC and the nitrogen atom (partial positive charge) of the indole moiety TRP391, were also evident. The π-donor H bond interactions are bonds that occur between H bond donor atoms and a π-ring that functions as an H bond acceptor, whereas π-cation interactions exist between a positively charged atom and the electrons of a delocalized π-system. The complex also involved three π-alkyl hydrophobic interactions. The AITC-ODR1 complex, according to the binding affinity, was found to be the second best. This complex consisted of two electrostatic bonds (one attractive charge and one π-cation type), two H bonds (one conventional and one C–H bond), and two hydrophobic bonds (both alkyl) (Figure 8B). The AITC-nGPCR complex consisted of two H bonds (both C–H) and two hydrophobic bonds (both alkyl) (Figure 8C).

**Figure 9:**
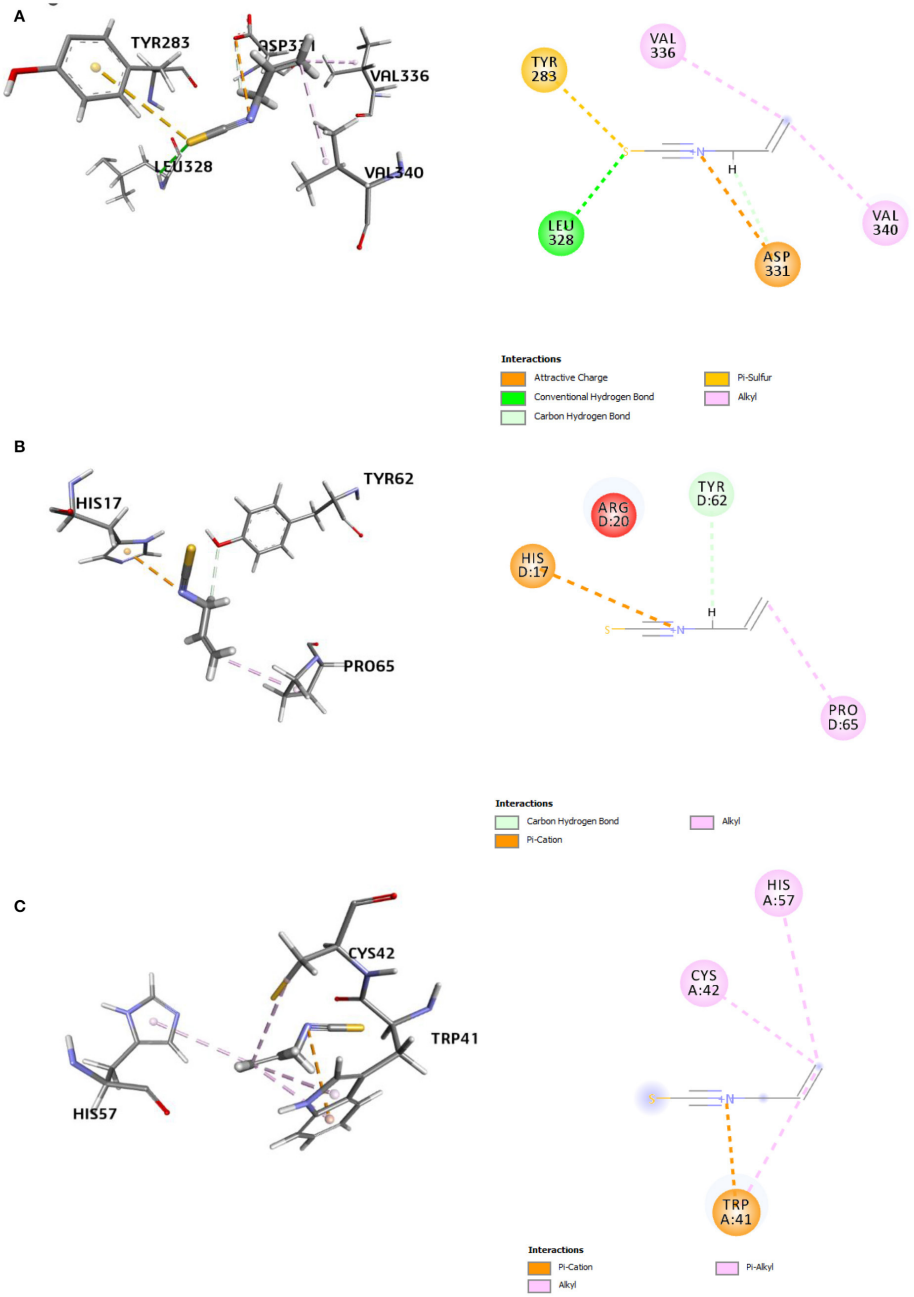
3D and 2D representations of the AITC-bound target sites of **(A)** chitin synthase (CS), **(B)** 2,3-dihydroxy benzoic acid decarboxylase (6m53), and **(C)** trypsinase (1try).

Eight receptor proteins of Fol were screened against AITC (Supplementary Figure 3). The binding energies of the screened AITC-target complexes are given in Table 4. The *in silico* activity varied in decreasing order as: AITC-CS > AITC-6m53 > AITC-1try > AITC-6fat > AITC-5jrm > AITC-4ovw > AITC-5ajh > AITC-5od4. The most favorable docking and binding energy value for the AITC-CS complex may be attributed to the dual-pronged factors, viz., the quantum of favorable hydrogen bonding and the specificity of the AITC binding to the Chitin Synthase 1 motif (Supplementary Figure 4). The aforementioned factors were further augmented by the presence of favorable electrostatic bonding between the partial positive charge on the nitrogen of –N–C=S moiety of AITC and the negatively charged oxygen moiety of the ASP331 residue (Table 5, Figure 9A). Additionally, the five different types of thermodynamically stable interactions, viz., electrostatic interaction, conventional hydrogen bonding, carbon-hydrogen bonding, alkyl hydrophobic bonding, and π-sulfur bond (between the partial negatively charged sulfur atom of AITC and the π-ring of the aromatic amino acid residue TYR283), quite evidently contributed. The most favorable interaction between AITC and the Chitin Synthase1 binding domain of CS (Figure 9A) suggested a possible inhibition of fungal cell wall biosynthesis and retarded hyphal growth and differentiation.

**Table 4:** Molecular docking results of AITC and the *Fusarium oxysporum* target proteins studied *in silico*.

**Protein target**	**Enzyme**	**Binding energy (kcal mol^**−1**^)**	**Hbond**	**Hphob**	**VwInt**	**Eintl**	**Dsolv**
1try	Trypsin	−13.950	−1.87	−2.70	−13.56	1.062	4.337
4ovw	Endoglucanase	−12.385	0.00	−2.59	−13.29	0.393	2.130
5ajh	Cutinase	−11.258	−2.32	−2.89	−10.82	0.448	4.363
5jrm	Xylanase	−12.873	−2.27	−2.53	−13.40	0.453	4.232
5od4	Avr2 effector protein	−9.871	−1.71	−2.70	−10.65	2.410	4.549
6fat	Feruloyl esterase	−13.869	−1.32	−3.29	−12.07	0.781	3.137
6m53	2,3-Dihydroxybenzoic acid decarboxylase	−15.452	−3.95	−2.90	−13.43	0.863	5.037
CS	Chitin synthase	−15.459	−4.28	−2.76	−13.86	0.293	3.378

*Hbond, hydrogen bond energy; Hphob, hydrophobic energy in exposing a surface to water; VwInt, van der Waals interaction energy (sum of gc and gh van der waals); Eintl, internal conformation energy of the ligand; Dsolv, desolvation of exposed H-bond donors and acceptors. Lower values of all these parameters suggest better interactions*.

**Table 5:** Molecular interaction details between AITC and the *F. oxysporum* target proteins.

**Target protein**	**Bond between atoms**	**Distance**	**Category**	**Type of bonding**
CS	:AITC:N2 -:ASP331:OD2	4.64458	Electrostatic	Attractive charge
	:LEU328:HN -:AITC:S1	2.97866	Hydrogen Bond	Conventional H bond
	:AITC:H7 -:ASP331:OD2	2.85029	Hydrogen Bond	C-H bond
	:AITC:S1 -:TYR283	5.22553	Other	π-sulfur
	:AITC:C5 -:VAL336	4.86375	Hydrophobic	Alkyl
	:AITC:C5 -:VAL340	4.71655	Hydrophobic	Alkyl
5ajh	C:TYR120 -:AITC:C3	4.44057	Hydrophobic	π-alkyl
	C:HIS189 -:AITC:C3	4.62325	Hydrophobic	π-alkyl
4ovw	A:ARG285:HH22 -:AITC:S1	3.03934	Hydrogen Bond	Conventional H bond
	:AITC:H1 - A:LEU283:O	2.28529	Hydrogen Bond	C-H bond
	:AITC:C3 - A:ILE210	4.90725	Hydrophobic	Alkyl
	:AITC:C3 - A:ARG285	4.60916	Hydrophobic	Alkyl
	A:TRP241 -:AITC:C3	3.48665	Hydrophobic	π-alkyl
	A:TRP241 -:AITC:C3	4.11889	Hydrophobic	π-alkyl
1try	:AITC:N1 - A:TRP41	4.78526	Electrostatic	π-cation
	:AITC:C3 - A:CYS42	4.84342	Hydrophobic	Alkyl
	A:TRP41 -:AITC:C3	4.31032	Hydrophobic	π-alkyl
	A:TRP41 -:AITC:C3	4.01407	Hydrophobic	π-alkyl
	A:HIS57 -:AITC:C3	4.73545	Hydrophobic	π-alkyl
5od4	:AITC:C3 - A:ARG88	4.17728	Hydrophobic	Alkyl
6fat	:AITC:N1 - B:TYR351	4.10324	Electrostatic	π-cation
	:AITC:S1 - B:PHE349	5.28155	Other	π-sulfur
	:AITC:C3 - B:ALA227	4.31028	Hydrophobic	Alkyl
	:AITC:C3 - B:LEU233	5.40926	Hydrophobic	Alkyl
	B:PHE230 -:AITC:C3	5.18428	Hydrophobic	π-alkyl
6m53	:AITC:H2 - D:TYR62:OH	2.92361	Hydrogen Bond	C-H bond
	:AITC:N1 - D:HIS17	3.74886	Electrostatic	π-cation
	:AITC:C3 - D:PRO65	4.04372	Hydrophobic	Alkyl
5jrm	:AITC:N1 - A:GLU176:OE2	3.36493	Electrostatic	Attractive charge
	:AITC:H1 - A:GLU176:OE2	2.66816	Hydrogen Bond	C-H bond
	:AITC:S1 - A:TYR178	3.94673	Hydrogen Bond; Other	π-donor H bond; π-sulfur
	:AITC:C3 - A:PRO97	3.75203	Hydrophobic	Alkyl
	A:TYR95 -:AITC:C3	4.77124	Hydrophobic	π-alkyl

The second best AITC-receptor complex, according to the binding affinity, was the AITC-6m53 complex. This complex consisted of one electrostatic bond (π-cation type), one H bond (C–H bond), and one hydrophobic bond (alkyl type) (Table 5). Interactions with C–H bonds are called weaker H bonds where a polarized carbon atom is the donor. The interactions are illustrated in the 2D diagram of the complex (Figure 9B). The observed inhibition of the 6m53 target protein by AITC suggests that MEO has a role in the probable disruption of indole-catechol conversion. The third most inhibited complex, i.e., the AITC-1try complex (Figure 9C), consisted of one electrostatic bond (π-cation type) and four hydrophobic bonds (one alkyl and three π-alkyl type) (Table 5). This inhibition is significant in terms of the interference of AITC in the invasive strategy of Fol, since proteases like trypsin are a vital part of the pathogenesis process.

It is clear from the docking and simulation findings that the fungal inhibition by AITC, as represented by the *in silico* scores of AITC against the eight receptors, is a complex consequence of diversity in the quantum equilibria of the studied interactions.

## Discussion

The GC-MS analysis established AITC as the major component of MEO, which is similar to earlier reports (Mejía-Garibay et al., 2015; Reyes-Jurado et al., 2019). Allyl isothiocyanate has been recognized to be responsible for the potential bioactivity of MEO against different pests and pathogens. This volatile compound is produced as a result of the hydrolysis of glucosinolates by the endogenous enzyme myrosinase present in plants of the *Brassicaceae* family (Fenwick et al., 1983). It is a common practice to biofumigate the field soil for the management of soil-borne pests and diseases by incorporating the fresh plant parts (green manuring), dried plant parts, or seed meals of the *Brassica* species (Ntalli and Caboni, 2017; Dutta et al., 2019). The process of glucosinolate hydrolysis is a function of several factors, such as the state of incorporated plant parts, soil type, moisture content, and environmental factors (Price et al., 2005; Gimsing and Kirkegaard, 2009; Wang and Mazzola, 2019). Thus, MEO/AITC has been often reported to efficiently control many soil-borne pests and pathogens like *Ralstonia solanacearum, Rhizoctonia solani, Sclerotium rolfsii, F. oxysporum, Phytophthora infestans, Pythium aphanidermatum*, and *M. incognita* (Harvey et al., 2002; Dhingra et al., 2004; Aissani et al., 2013; Ren et al., 2018; de Carvalho Pontes et al., 2019, Yu et al., 2019).

This study inferred the LC_50_ values of MEO against Mi at 24, 48, and 72 h as 47.7, 30.3, and 20.4 μg ml^−1^, respectively. Earlier, Yu et al. (2005) reported the LC_50_ of pure AITC against Mi as 17 μg ml^−1^ after 24 h. In another study involving soil fumigation, the LC_50_ value of AITC against *Meloidogyne* spp. was found to be 18.05 mg kg^−1^ (Ren et al., 2018). Surprisingly, the most important finding of this study was that MEO showed immediate short-term nematostatic action against Mi even at a concentration as low as 1 μg ml^−1^, which dramatically shifted to show nematicidal effect for a longer period of exposure at higher concentration. Recently, a similar observation was made by Dahlin and Hallmann (2020) during an *in vitro* bioassay of various isocyanates against *M. hapla*, where the significant recovery of isocyanate-treated J_2_s from inactive states was observed at a lower concentration. The same study reported that the J_2_s exposed to a low concentration of AITC, after recovery, were still able to cause root galling in cucumbers. However, this study reported contrasting observations. This study demonstrated that both time of exposure and concentration of MEO administered provided significant effects on the infectivity potential of treated *Meloidogyne* juveniles. Increase in exposure time, irrespective of concentration, reduced the potency of revived nematodes. Microscopic images of infected roots confirmed these findings. For the first time, this study also dwelled upon the permeation of essential oil (MEO in this study) inside the nematode body through the cell membrane.

*An in silico* molecular modeling study in this study was attempted to understand and unravel the basis of the behavioral response of *M. incognita* toward MEO exposure. The favorable docking scores of AITC against all seven putative target proteins pointed toward the possible multi-modal inhibitory action of MEO. The highly favorable interactions of AITC with the three target proteins, namely, AChE, ODR1, and nGPCR, suggested that AITC might affect the neurotransmission and chemosensing functions of *M. incognita* (Bresso et al., 2019). This observation justified the nematostatic effect of MEO and the reduced infectivity potential. Due to the volatile nature of the compound, there was a revival of the nematodes at a low concentration of test emulsions; however, at high concentrations with longer periods of exposure, the multi-modal action of AITC became evident due to nematode death. Such a detailed *in silico* investigation of the mode of action of AITC on root-knot nematodes (*M. incognita*) is being reported for the first time.

Very low EC_50_ values of 6.42 μg ml^−1^ and an MIC of 256 μg ml^−1^ were obtained for MEO during an *in vitro* bioassay against Fol. Previous studies documented the bioactivity of AITC against *Fusarium* spp. at an even lower concentration. Cardiet et al. (2012) reported the IC_50_ and MIC of AITC as 1.35 μl L^−1^ and 19.8 μl, respectively, against *F. graminearum*. Recently, Ren et al. (2018) also reported the EC_50_ value of AITC as 0.95 mg L^−1^ against *F. oxysporum* in a fumigation bioassay. It is noteworthy that the TOFU-IHBT isolate used in this study was one of the most virulent strains of Fol (Sidharthan et al., 2018). In this regard, MEO exhibited promising potential against this hard-to-manage strain of Fol, as the MEO test emulsion performed better than commercial formulation. To understand the mechanism of this action, an *in silico* molecular docking study provided interesting and novel leads. The binding energies provided a clear insight into the role of AITC in inhibiting the functions of Fol as a plant pathogenic fungus. The binding energy range (−9.8 to −15.5 kcal mol^−1^) pointed toward the possible multi-modal inhibitory action of AITC on Fol, as the target proteins encompass a diverse range of molecular and biological functions in enabling the fungus to attack the plant. To the best of our information, this report is the first of its kind to highlight the mode of action of AITC present in mustard essential oil. In a recent report by Li et al. (2020), AITC was reported to act upon a yeast-like vacuolar transient receptor potential channel regulator (FsYvc1) in *F. solani*. Similarly, Mohamed et al. (2019) also hinted that AITC might be responsible for endopolygalacturonase enzyme inhibition in the case of *F. oxysporum*.

## Conclusions

This study specifically aimed to understand the potential of MEO against *M. incognita* and *F. oxysporum*. It clearly established the exceptionally high potential of MEO against these two organisms and confirmed that the multimodal target protein interactions were responsible for the observed responses. Findings reported herein will be further subjected to confirmation by wet laboratory molecular studies to develop ready-to-use MEO-based multicomponent formulations augmented with other bioactives for the simultaneous management of the root-knot nematode-induced *Fusarium* wilt disease complex in agricultural crops.

## Data Availability Statement

The original contributions presented in the study are included in the article/Supplementary Material, further inquiries can be directed to the corresponding author/s.

## Author Contributions

AD: investigation, methodology, formal analysis, writing—original draft, and visualization. AM: investigation, methodology, validation, and formal analysis. AK: validation, formal analysis, and writing—review and editing. MM and AC: investigation. MK and VS: methodology and supervision. UR and PS: resources. SS: resources and validation. NP: validation. RK: supervision. AK and SD: data curation. AS: conceptualization, supervision, project administration, funding acquisition, and writing—review and editing. All authors contributed to the article and approved the submitted version.

## Conflict of Interest

The authors declare that the research was conducted in the absence of any commercial or financial relationships that could be construed as a potential conflict of interest.

## Publisher's Note

All claims expressed in this article are solely those of the authors and do not necessarily represent those of their affiliated organizations, or those of the publisher, the editors and the reviewers. Any product that may be evaluated in this article, or claim that may be made by its manufacturer, is not guaranteed or endorsed by the publisher.
