# Supplementary material for: Deciphering the Behavioral Response of Meloidogyne incognita and Fusarium oxysporum Toward Mustard Essential Oil
Source: Front Plant Sci. 2021 Aug 26;12:714730. doi: 10.3389/fpls.2021.714730 (PMC8427441; doi:10.3389/fpls.2021.714730)

**Supporting information**


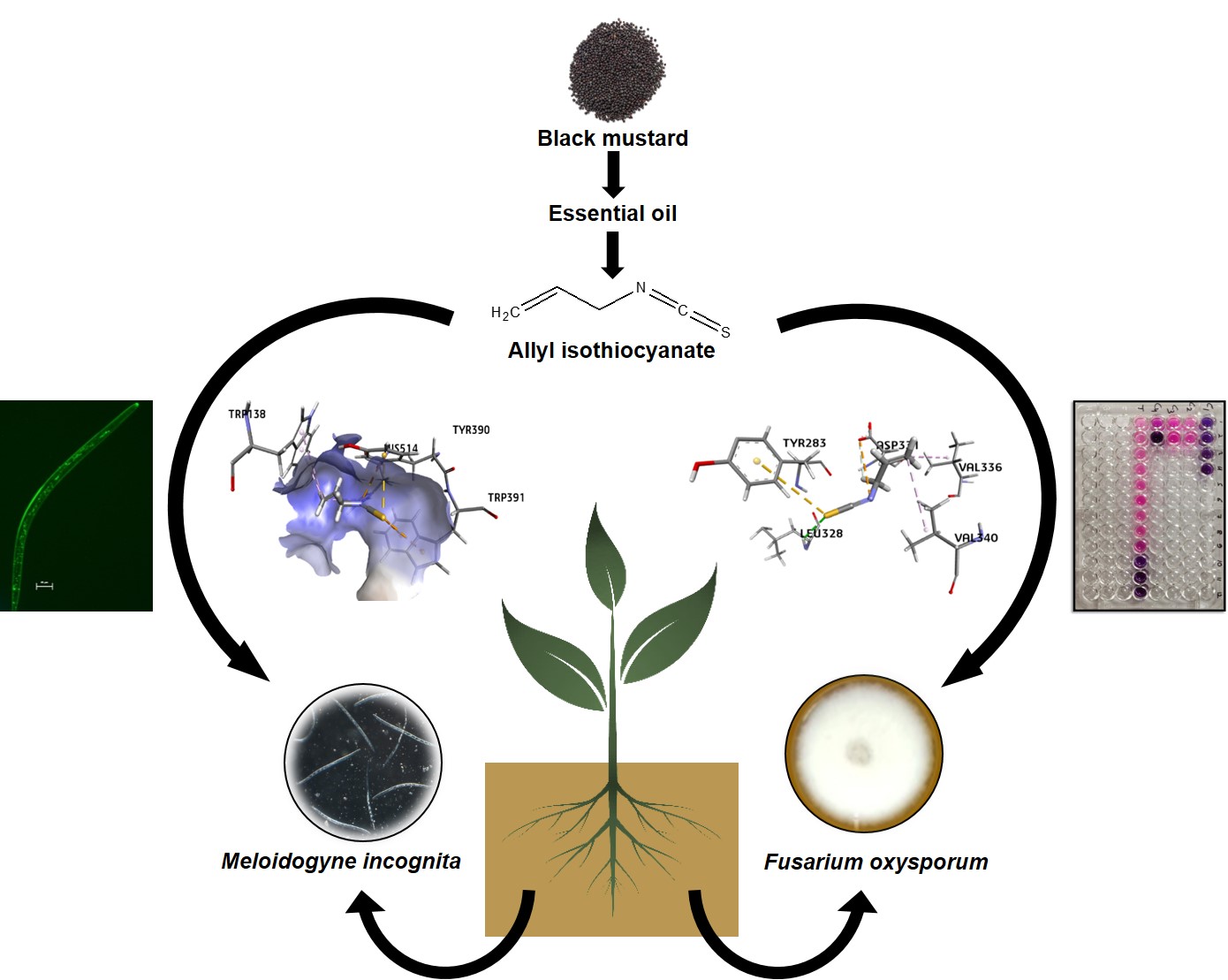


**GRAPHICAL ABSTRACT**

**S1 Table 1. Target sequences screened for *in silico* nematicidal activity**

| **Receptor** | **Amino Acid Sequence** | **Source** |
| --- | --- | --- |
| Cytochrome-c oxidase subunit 1 | LVTKSVTHKNIGFIYLFFSFWSGLMGLSLSMLLRMDLMKSGMVIGDGQLYNVILTSHALVMIFFMVMPGLIGGFGNFFFPILINCIDLFLPRVNNMSYWFLPGSLILLMFSLFMDKGSGTGWTLYPPLMIDGQPGRSTDLVIFSLHFSGISSISSGINFLSTCHEMRLEVKTLEIMSLFVWCLIITVFLLVLSLPVLASGITMGLSDRNFNTGFFDSNMGGNILMFQHLFWFFGHPEVYVLIAPAFGLVSMVMVLLSSKKDLYGRKGMILAIMSIGFIGCLVWGHHMFTVGMDHDSRAYFSSATMIIAIPTGMKIFSWMMTLYGSKLNWNYLILWIMGFIFMFTVGGLSGLILSNAGLDIFLHDTYYVVAHFHYVLSMGAVFGIFLGFFFSYGFMFGLMMNSVLVKSFFYIFFLGVNLTFFPMHFSGLQGQPRKYMSYSSDYLFWQMFASIGSLLSLFSIFLLIYLILESMIIFRLLIFDLFSFSMVSLNVNNYFHTNLDLSMIWLK | NCBI GenBank |
| AChE | MRKRRRKTTAFSINTSELLRLYFKFSSHSCLTFIFCCFFCLIVYCSSVHGRSSPVALTDVLIQTTLGKIIGFKQKFDGKSVHTFLGVPYAKSPTGSGRFGLPEMIEPWEGEFRADKPARTCFFSRDTMFPDFPGAEMWNPPNDIDEDCLAMNIWVPEHHDGTVLVWIYGGGFYSGSPSLDLYDGRVLAVQERAVVININYRLGPFGFLYFGDDTSVPGNMGLQDQQMALKWIHEHIAHFGGDPRRVTLFGESAGSASAMAHMFADGSYSLFSRIIAQSGSIINNWATKPKASILQISLQLAHHLNCSNGNNSTKAMQNIVECIRRVPTSIIQRAGDAVSQSLSLPMDFAFVPIDEDTHFFRGNVFDKLRRKNFKRDVSILVGTVRDEGTYWLPYCLQKNGFGFNHTISPEDHINQALISETDYTKAFDAFLPYFGNSNLVRHALMHAYSHLPTEKQEQRWRDGVARFLGDYFFTCDSIEFADIVSDELYGSVYSFYFTRRSSANPWPQWMGAMHGYEIEYVFGLPLRSPHLYDPSELELEISFSTKIMEFWGHFARTGEPVEFWPKYNRITRKSLVLSEEIATGTSHRIYVDVHGKLCRLLEEAQAVAGITGEQRSRICPDGRATTVNYGQEISMEDVKEEMQLNRGISGINRIPSIKIYISLIILSLALLRSPEISFLYSSFIFK | NCBI GenBank |
| Hsp90 | MSLIINTFYSNKEIFLRELISNSSDALDKIRYQALTDPAQLETGKDLYIKIVPNKADKTLTIMDTGVGMTKADLVNNLETIAKSGTKAFMEALQAGADISMIGQFGVGFYSAFLVADRVTVTSEHNDDDCHQWESSAGGSFIIRNCVDPEMTRGTKITLYLKEDQTDYLEERRIREVVKKHSQFIGYPIKLLVEKERDKEISDDEAEDEKKDVKKEEEKEEEKEIKKEEGEDKEGEDEDKDKKDGEKKKKTKKIKEKYTEDEELNKTKPIWTRNPDDITNEEYAEFYKSLSNDWEDHLAVKHLSVEGQLEFRALLFVPQRAPFDMFENKKQKNAIKLYVRRVFIMENCEELMPEYLNFIKGVVDSEDLPLNISREMLQQSKILKVIRKNLVKKCIELFDEIAEDKDNFKKFYEQFSKNLKLGIHEDSVNRKKLAEYLRYNTSSSGDELVSLKDYVGRMKENQTCIYYITGESKEVVQNSAFVERVKKRGFEVIYMVDPIDEYCIQQLKEFDGKKLVSVTKEGLELPESEEEKKKFEEDKVKFEKLCKVIKDILDKKVQKVSVSNRLVSSPCCIVTGEYGWTANMERIMKAQALRDSSTMGYMASKKNLEINPDHSIIKSLRERIDSDQDDKTAKDLVVLLYETALLTSGFSLEDPQQHASRIYRMVKLGLDITEEDLEGGEQQPCTSGEPVEKIAGAEEDASRMEEVD | NCBI GenBank |
| ODR1 | MMTGQQSTESFLATLAIYNACYGFCLGSSLTSTGSFASDPNNPAFVANLRGKSFQGIKKFLLPKRNFQFKGSFGQVNLTSWPAPLQNLAIYTLPSSGGQYSLIYTAISIPSSSCGTFECFDIQLQTSPNISEDLLWQKQCSNTIPSCIYSGGCSSLVPYFSAGAAIVLVAAAAGIVYTIQRKKRLDVFRVHWRIGRQQFKVIENKQAKGKATGIGQEGAWSKRRQLHAYALIGTNKAEFIVLRQMKKIYWDKIELHFIFELKKLNHDNLTTFMGICYNDGDKFYVCHSLVERGTLEDYIHDLDFQLDNTFRSAFLRDILKGVKYLHKSSIGYHGMLNLQNVLIDSNWVLKLTNFGIGNLLNRAIRREQLQLIELIPLNTYLTVAPENLIDISYGREYPNGTTIGDIYSMGMVMYHILFRLAPYERTTLSPKEVIDQVRQHNLKPILENTLPEEKPLVDAMEQCWQKNLDLRPRLRQLAQVVSTVFQASQGNLIDQMRRMNEKHALNLEKLVTQRNAELAQAREQTERLLNEMLPPSIAAQLKEHKSVEPRSYDSATVLFCQLVDFSTVLSKFPPDQVIDFLNQVFSTFDTIIRNHDAYKVETTGETYMVASGVPNENENRHVFEISEVAMEFREVSYTYKSINFPDWKLQLRIGYHCGPIAAGVIGIKAPRYCLFGDTVNFASRMQSNAAPNQIQMSESTALLLMGVSKYKLTKRGIVKVKGKER | WormBase ParaSite Database |
| ODR3 | SCQSEEVREQLSKNKAIEKQLTSDRRAASSIIKLLLLGAGECGKSTVLKQMQILHSNGFTEEEINERKAVVYSNTVTSMAAILKAMDNVLHMPMDDASKERDRNLIFRAIENGEENLPFTDPIAKALQNLWGDKAVKKAYEMRSEYQLNDSAKYFLDSVSRIHEPGYRPTEQDILYSRVATTGVVEVKFIIKGNMEFRVFDVGGQRSERRKWIHCFDNVEAIIFITAISEYDQVLFEDETTNRMIESMQLFSSICNSSWFLNTAMILFLNKKDLFLEKIQRVNITTCFPDYEGSQNYEEAVNFIKMKFAELNQHPDKKTIYMHETCATDTN | WormBase ParaSite Database |
| Neuropeptide GPCR | MVSSISLNQQINQIEIENCIELNSVLDQFGDWTLRLDVKFFYSLFYAAIFIVGLIGNGFLVGTIRRRMTVANVFLMNLAISDLLLCITALPITPVLAFVKRWIFGLALCKLVPLCQGISVLISSYCLCLIAVDRYRSIVTPLKVPWNIQXAQWLMTLCWTFCIIISSPLFIVQGLQQIVYKNMTFCGEFCTELNWPTDFRIKLFYGISLLSIQFLIPTLIMTYCYWKILQKVRQDWLVPTNNSIMSLEQQAQTAIRKRRVMYVLILMVLIFMGSWMPLTFVNLLRDIGISFLETQMYFKLLNVXAVAMTSVVSNPLLYFYMSKRXRRALRDDMYWLTNARRQQNQXVGGLLAKFTPSPSIGLLYKKSLERHILQNATAKYNPYRRGTLADPTTLGREKVLQEMHANCFLLVPLMPLCVANQQRLATNQREISNNNNINLNFKRQKHPKFVCEA | NCBI GenBank |
| CLAVATA3/ESR (CLE)-related protein | MFTNSIKNLIIYLMPLMVTLMLLSVSFVDAGKKPSGPNPGGNN | UNIPROT Database |

*Meloidogyne incognita* acetyl cholinesterase (AChE), *M. incognita* Heat Shock Protein 90 (Hsp90), *M. incognita* odorant response gene-1 (ODR1), *M. incognita* odorant response gene-3 (ODR3) and *M. incognita* neuropeptide G-Protein Coupled Receptor (nGPCR)

**S1 Fig 1.** Quality assessment of the Chitin Synthase secondary structure homology model using A) Statistical Z-score and B) Ramachandran Plot


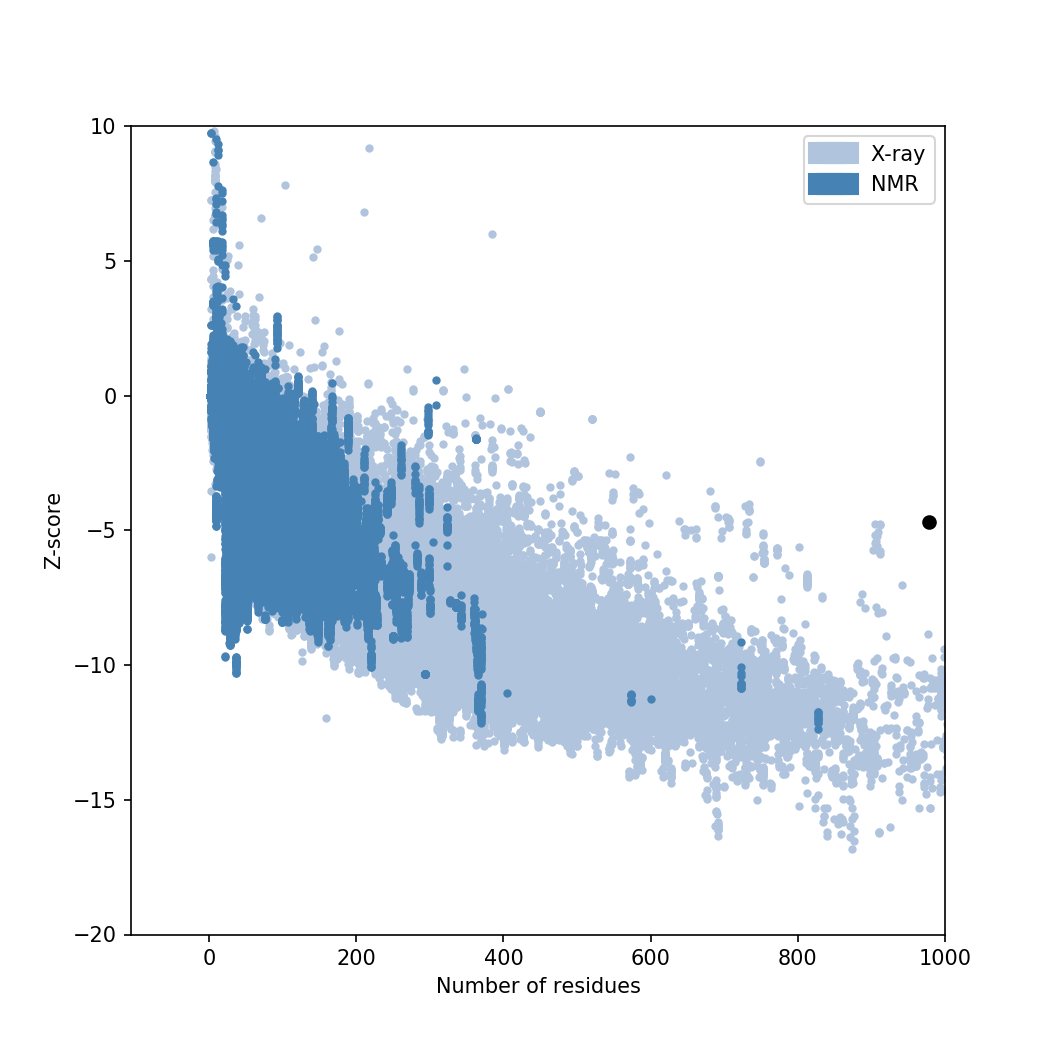


**Z-score =-4.7**


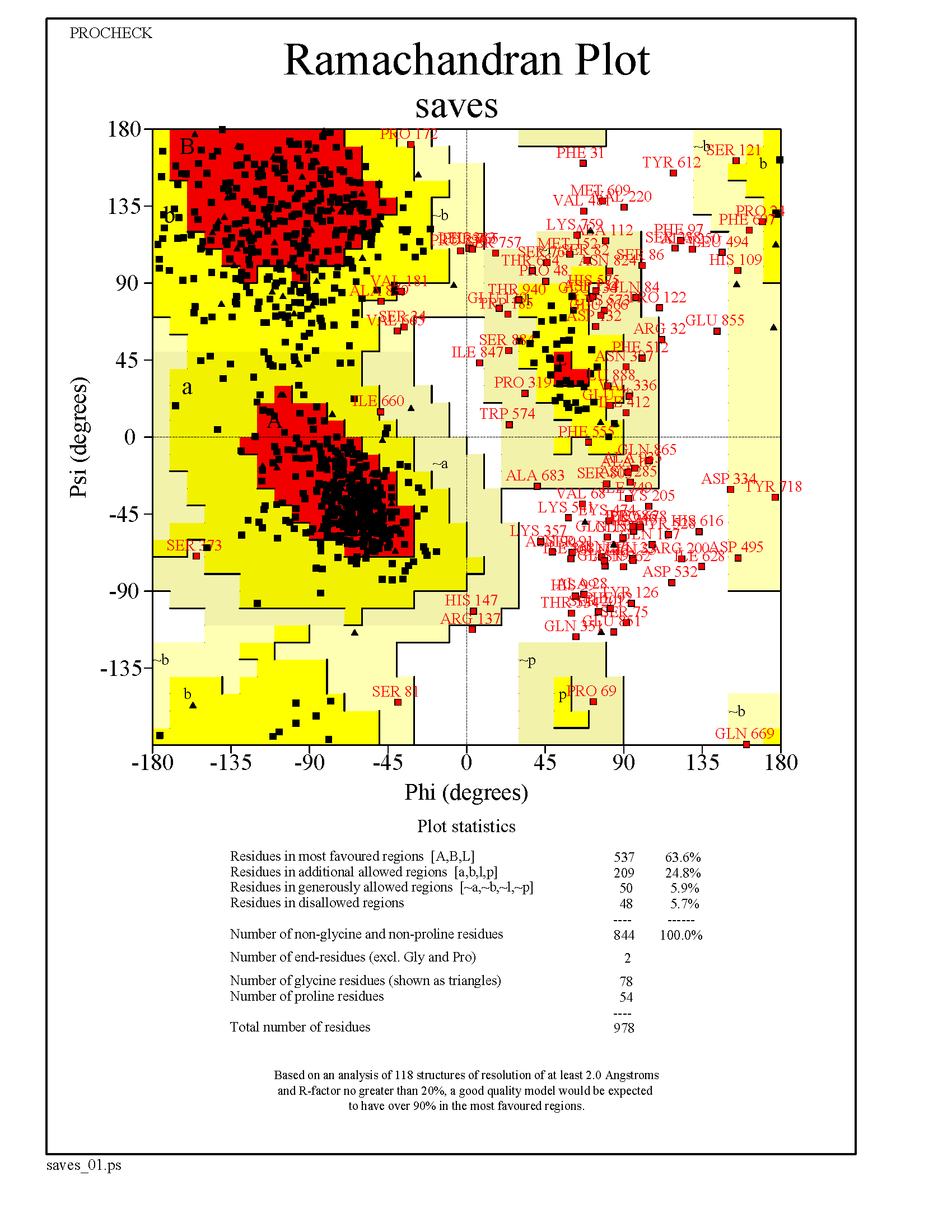


**A**

**B**

**S2 Fig 2.** Bioassay of MEO against *F. oxysporum* f. sp. *lycopersici* isolate TOFU-IHBT by poisoned food assay. 1^st^ row: i) 50 μg mL^-1^ ii) 100 μg mL^-1^ iii) 250 μg mL^-1^ iv) 500 μg mL^-1^ v) 1000 μg mL^-1^; 2^nd^ row: i) 1 μg mL^-1^ ii) 3.12 μg mL^-1^ iii) 6.25 μg mL^-1^ iv) 12.5 μg mL^-1^ v) 25 μg mL^-1^; 3^rd^ row: i) water control ii) surfactant control iii) positive control with Nativo 1 μg mL^-1^ iv) positive control with Nativo 50 μg mL^-1^


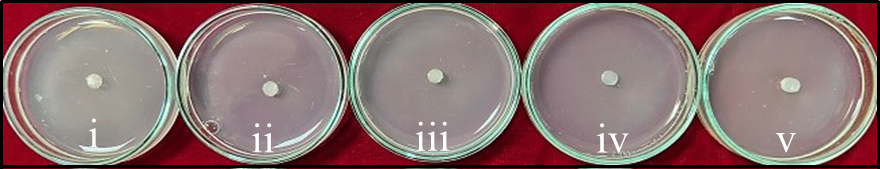

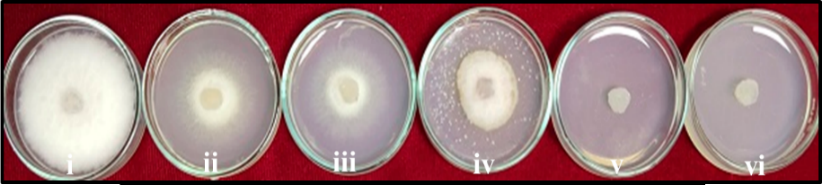

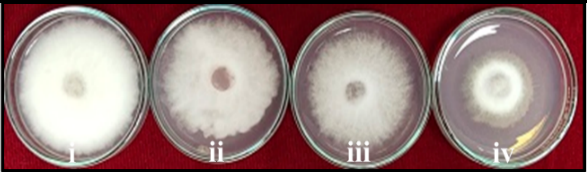


**S3 Fig 3.** 3-D structure of putative target sites of *Fusarium oxysporum*: A) 1try (trypsinase), B) 4ovw (endoglucanase), C) 5ajh (cutinase), D) 5jrm (xylanase), E) 5od4 (Avr2 effector protein), F) 6fat (feruloyl esterase), G) 6m53 (2,3-dihydroxy benzoic acid decarboxylase), H) CS (chitin synthase)


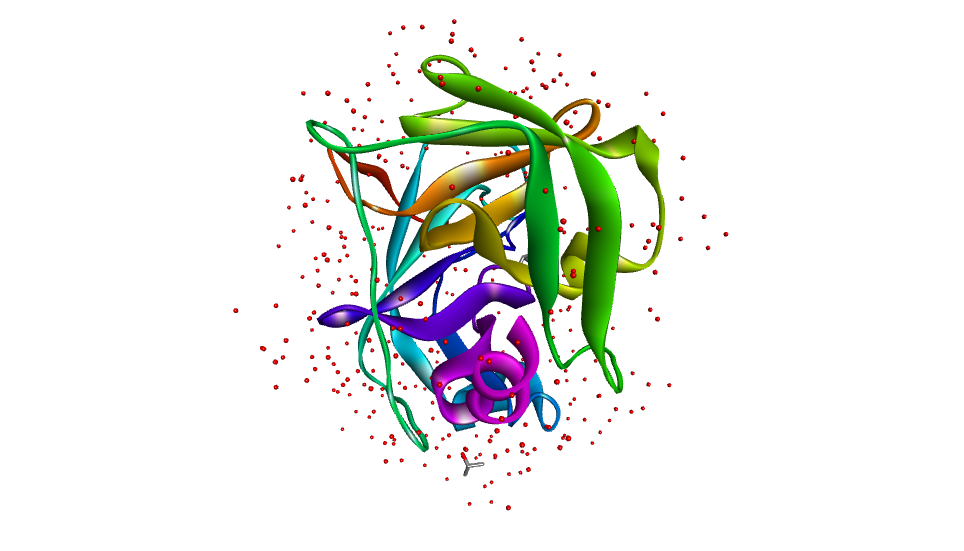

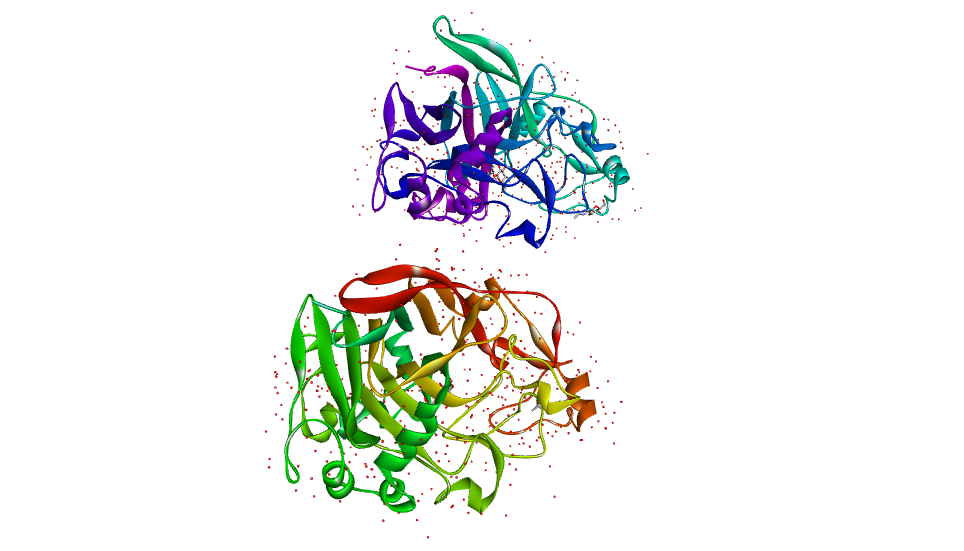

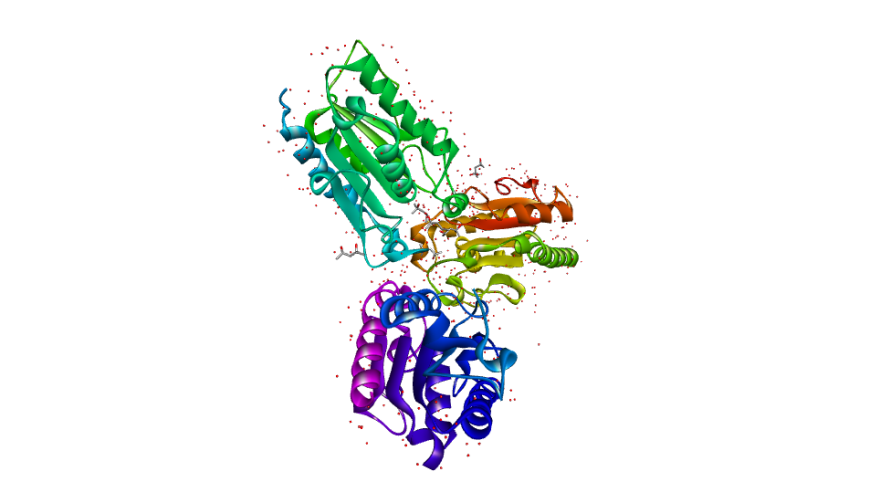


**(A)**


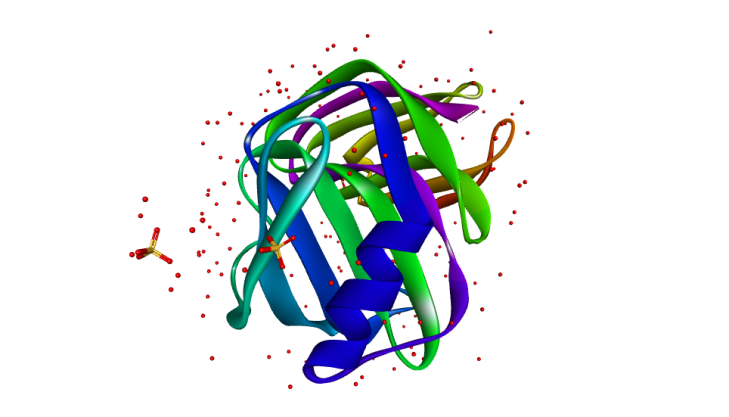

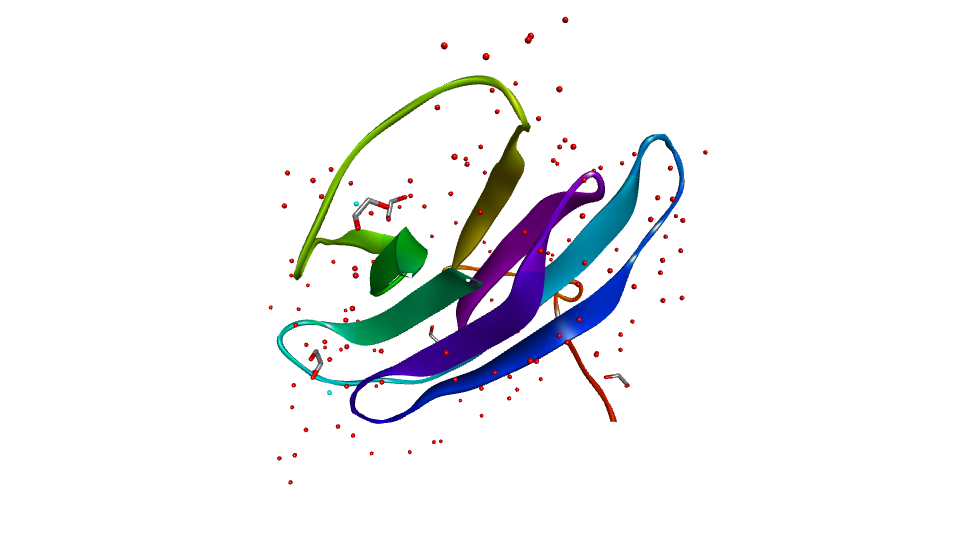

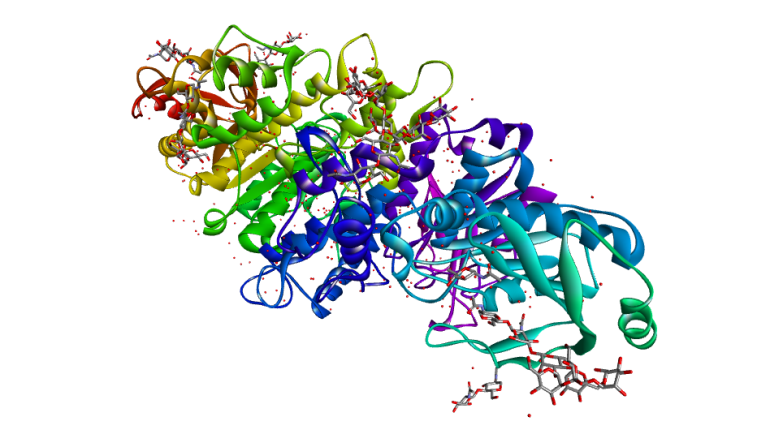

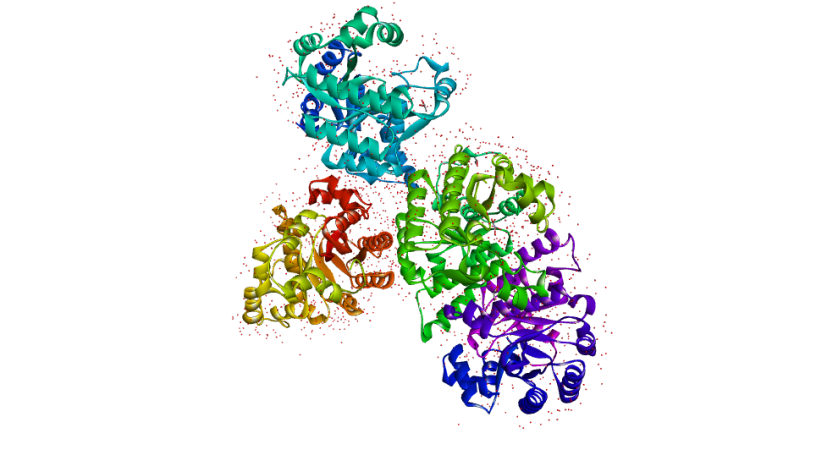

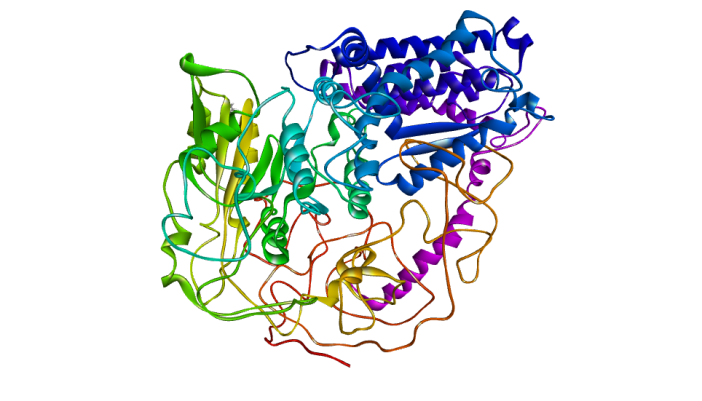


**(B)**

**(C)**

**(D)**

**(E)**

**(F)**

**(G)**

**(H)**

**S4 Fig 4.** Chitin Synthase (CS): Motifs, Chitin Synthase 1 motif highlighted in the sequence and in the energy minimized structure of the modelled protein (in yellow)


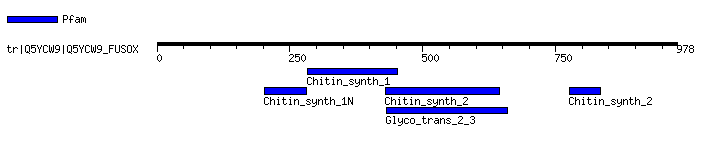

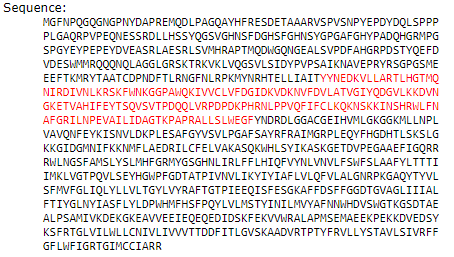

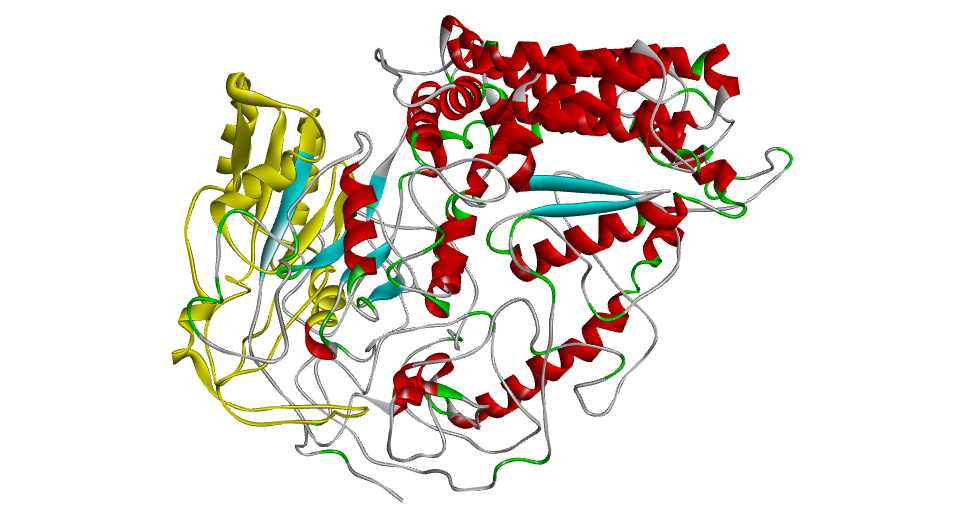

Supplement: Supplementary file 1 [file Data_Sheet_1.docx]
